# Digitally‐enabled, patient‐centred care in rhinitis and asthma multimorbidity: The ARIA‐MASK‐air^®^ approach

**DOI:** 10.1002/clt2.12215

**Published:** 2023-01-07

**Authors:** Jean Bousquet, Josep M. Anto, Bernardo Sousa‐Pinto, Wienczyslawa Czarlewski, Anna Bedbrook, Tari Haahtela, Ludger Klimek, Oliver Pfaar, Piotr Kuna, Maciej Kupczyk, Frederico S. Regateiro, Boleslaw Samolinski, Arunas Valiulis, Arzu Yorgancioglu, Sylvie Arnavielhe, Xavier Basagaña, Karl C. Bergmann, Sinthia Bosnic‐Anticevich, Luisa Brussino, G. Walter Canonica, Victoria Cardona, Lorenzo Cecchi, Claudia Chaves‐Loureiro, Elisio Costa, Alvaro A. Cruz, Bilun Gemicioglu, Wytske J. Fokkens, Juan Carlos Ivancevich, Helga Kraxner, Violeta Kvedariene, Désirée E. Larenas‐Linnemann, Daniel Laune, Renaud Louis, Michael Makris, Marcus Maurer, Erik Melén, Yann Micheli, Mario Morais‐Almeida, Joaquim Mullol, Marek Niedoszytko, Yoshitaka Okamoto, Nikolaos G. Papadopoulos, Vincenzo Patella, Nhân Pham‐Thi, Philip W. Rouadi, Joaquin Sastre, Nicola Scichilone, Aziz Sheikh, Mikhail Sofiev, Luis Taborda‐Barata, Sanna Toppila‐Salmi, Ioanna Tsiligianni, Erkka Valovirta, Maria Teresa Ventura, Rafael José Vieira, Mihaela Zidarn, Rita Amaral, Ignacio J. Ansotegui, Annabelle Bédard, Samuel Benveniste, Michael Bewick, Carsten Bindslev‐Jensen, Hubert Blain, Matteo Bonini, Rodolphe Bourret, Fulvio Braido, Pedro Carreiro‐Martins, Denis Charpin, Ivan Cherrez‐Ojeda, Tomas Chivato, Derek K. Chu, Cemal Cingi, Stefano Del Giacco, Frédéric de Blay, Philippe Devillier, Govert De Vries, Maria Doulaptsi, Virginie Doyen, Gérard Dray, Jean‐François Fontaine, R. Maximiliano Gomez, Jan Hagemann, Enrico Heffler, Maja Hofmann, Ewa Jassem, Marek Jutel, Thomas Keil, Vicky Kritikos, Inger Kull, Marek Kulus, Olga Lourenço, Eve Mathieu‐Dupas, Enrica Menditto, Ralph Mösges, Ruth Murray, Rachel Nadif, Hugo Neffen, Stefania Nicola, Robyn O’Hehir, Heidi Olze, Yuliia Palamarchuk, Jean‐Louis Pépin, Benoit Pétré, Robert Picard, Constantinos Pitsios, Francesca Puggioni, Santiago Quirce, Filip Raciborski, Sietze Reitsma, Nicolas Roche, Monica Rodriguez‐Gonzalez, Jan Romantowski, Ana Sá‐Sousa, Faradiba S. Serpa, Marine Savouré, Mohamed H. Shamji, Milan Sova, Annette Sperl, Cristiana Stellato, Ana Todo‐Bom, Peter Valentin Tomazic, Olivier Vandenplas, Michiel Van Eerd, Tuula Vasankari, Frédéric Viart, Susan Waserman, Joao A. Fonseca, Torsten Zuberbier

**Affiliations:** ^1^ Institute of Allergology Charité – Universitätsmedizin Berlin Corporate Member of Freie Universität Berlin and Humboldt‐Universität zu Berlin Berlin Germany; ^2^ Fraunhofer Institute for Translational Medicine and Pharmacology ITMP Allergology and Immunology Berlin Germany; ^3^ University Hospital Montpellier Montpellier France; ^4^ Inserm Equipe d’Epidémiologie Respiratoire Intégrative CESP Villejuif France; ^5^ ISGlobal Barcelona Institute for Global Health Barcelona Spain; ^6^ IMIM (Hospital del Mar Medical Research Institute) Barcelona Spain; ^7^ Universitat Pompeu Fabra (UPF) Barcelona Spain; ^8^ CIBER Epidemiología y Salud Pública (CIBERESP) Barcelona Spain; ^9^ MEDCIDS ‐ Department of Community Medicine, Information and Health Decision Sciences Faculty of Medicine University of Porto Porto Portugal; ^10^ CINTESIS – Health Research Network Faculty of Medicine, University of Porto Porto Portugal; ^11^ RISE – Health Research Network, MEDCIDS Faculty of Medicine, University of Porto Porto Portugal; ^12^ Medical Consulting Czarlewski Levallois France; ^13^ MASK‐air Montpellier France; ^14^ Skin and Allergy Hospital Helsinki University Hospital University of Helsinki Helsinki Finland; ^15^ Department of Otolaryngology, Head and Neck Surgery Universitätsmedizin Mainz Mainz Germany; ^16^ Center for Rhinology and Allergology Wiesbaden Germany; ^17^ Section of Rhinology and Allergy Department of Otorhinolaryngology, Head and Neck Surgery University Hospital Marburg Philipps‐Universität Marburg Marburg Germany; ^18^ Division of Internal Medicine, Asthma and Allergy Barlicki University Hospital Medical University of Lodz Lodz Poland; ^19^ Allergy and Clinical Immunology Unit Centro Hospitalar e Universitário de Coimbra Coimbra Portugal; ^20^ ICBR, Coimbra Institute for Clinical and Biomedical Research, CIBB Faculty of Medicine University of Coimbra Coimbra Portugal; ^21^ Institute of Immunology Faculty of Medicine University of Coimbra Coimbra Portugal; ^22^ Department of Prevention of Environmental Hazards, Allergology and Immunology Medical University of Warsaw Warsaw Poland; ^23^ Institute of Clinical Medicine and Institute of Health Sciences Vilnius Lithuania; ^24^ Medical Faculty of Vilnius University Vilnius Lithuania; ^25^ Department of Pulmonary Diseases Celal Bayar University, Faculty of Medicine Manisa Turkey; ^26^ GRADeS Occitanie Toulouse France; ^27^ Quality Use of Respiratory Medicine Group Woolcock Institute of Medical Research The University of Sydney Sydney NSW Australia; ^28^ Sydney Local Health District Sydney NSW Australia; ^29^ Department of Medical Sciences Allergy and Clinical Immunology Unit University of Torino Torino Italy; ^30^ Mauriziano Hospital Torino Italy; ^31^ Department of Biomedical Sciences Humanitas University, Pieve Emanuele Milan Italy; ^32^ Personalized Medicine, Asthma and Allergy Humanitas Clinical and Research Center IRCCS Rozzano Italy; ^33^ Allergy Section Department of Internal Medicine Hospital Vall d'Hebron Barcelona Spain; ^34^ ARADyAL Research Network Barcelona Spain; ^35^ SOS Allergology and Clinical Immunology USL Toscana Centro Prato Italy; ^36^ Pneumology Unit Hospitais da Universidade de Coimbra Centro Hospitalar e Universitário de Coimbra Coimbra Portugal; ^37^ UCIBIO, REQUINTE Faculty of Pharmacy and Competence Center on Active and Healthy Ageing University of Porto (Porto4Ageing) Porto Portugal; ^38^ Fundaçao ProAR Federal University of Bahia and GARD/WHO Planning Group Salvador Bahia Brazil; ^39^ Cerrahpasa Faculty of Medicine Department of Pulmonary Diseases Istanbul University‐Cerrahpasa Istanbul Turkey; ^40^ Department of Otorhinolaryngology Amsterdam University Medical Centres, location AMC Amsterdam the Netherlands; ^41^ Servicio de Alergia e Immunologia Clinica Santa Isabel Buenos Aires Argentina; ^42^ Department of Otorhinolaryngology, Head and Neck Surgery Semmelweis University Budapest Hungary; ^43^ Faculty of Medicine Institute of Clinical Medicine Clinic of Chest Diseases and Allergology Vilnius University Vilnius Lithuania; ^44^ Faculty of Medicine Department of Pathology Institute of Biomedical Sciences Vilnius University Vilnius Lithuania; ^45^ Center of Excellence in Asthma and Allergy Médica Sur Clinical Foundation and Hospital México City Mexico; ^46^ KYomed INNOV Montpellier France; ^47^ Department of Pulmonary Medicine CHU Liege Liège Belgium; ^48^ GIGA I3 Research Group University of Liege Liège Belgium; ^49^ Allergy Unit “D Kalogeromitros” 2nd Department of Dermatology and Venereology National & Kapodistrian University of Athens “Attikon” University Hospital Athens Greece; ^50^ Sach's Children and Youth Hospital Södersjukhuset Stockholm Sweden; ^51^ Department of Clinical Science and Education Södersjukhuset Karolinska Institutet Stockholm Sweden; ^52^ Allergy Center CUF Descobertas Hospital Lisbon Portugal; ^53^ Rhinology Unit & Smell Clinic ENT Department Hospital Clínic Barcelona Spain; ^54^ Clinical & Experimental Respiratory Immunoallergy IDIBAPS, CIBERES University of Barcelona Barcelona Spain; ^55^ Department of Allergology Medical University of Gdańsk Gdansk Poland; ^56^ Chiba University Hospital Chiba Japan; ^57^ Chiba Rosai Hospital Chiba Japan; ^58^ Allergy Department 2nd Pediatric Clinic University of Athens Athens Greece; ^59^ Division of Allergy and Clinical Immunology Department of Medicine “Santa Maria della Speranza” Hospital, Battipaglia Salerno Italy; ^60^ Agency of Health ASL Salerno Italy; ^61^ Ecole Polytechnique Palaiseau IRBA (Institut de Recherche Bio‐Médicale des Armées) Bretigny France; ^62^ Department of Otolaryngology, Head and Neck Surgery Eye and Ear University Hospital Beirut Lebanon; ^63^ Department of Otorhinolaryngology, Head and Neck Surgery Dar Al Shifa Hospital Salmiya Kuwait; ^64^ Fundacion Jimenez Diaz, CIBERES Faculty of Medicine Autonoma University of Madrid Madrid Spain; ^65^ PROMISE Department University of Palermo Palermo Italy; ^66^ Usher Institute The University of Edinburgh Edinburgh UK; ^67^ Finnish Meteorological Institute (FMI) Helsinki Finland; ^68^ Department of Immunoallergology Cova da Beira University Hospital Centre Covilhã Portugal; ^69^ UBIAir ‐ Clinical & Experimental Lung Centre and CICS‐UBI Health Sciences Research Centre University of Beira Interior Covilhã Portugal; ^70^ International Primary Care Respiratory Group IPCRG Aberdeen Scotland; ^71^ Health Planning Unit Faculty of Medicine Department of Social Medicine University of Crete Crete Greece; ^72^ Department of Lung Diseases and Clinical Immunology University of Turku Turku Finland; ^73^ Terveystalo Allergy Clinic Turku Finland; ^74^ Unit of Geriatric Immunoallergology University of Bari Medical School Bari Italy; ^75^ University Clinic of Respiratory and Allergic Diseases Golnik Slovenia; ^76^ Faculty of Medicine University of Ljubljana Ljubljana Slovenia; ^77^ Department of Allergy and Immunology Hospital Quironsalud Bizkaia Bilbao Spain; ^78^ Université Paris‐Saclay, UVSQ University Paris‐Sud Villejuif France; ^79^ National Center of Expertise in Cognitive Stimulation (CEN STIMCO) Broca Hospital Paris France; ^80^ Mines ParisTech CRI ‐ PSL Research University Fontainebleau France; ^81^ University of Central Lancashire Medical School Preston UK; ^82^ Odense Research Center for Anaphylaxis (ORCA) Odense Denmark; ^83^ Department of Dermatology and Allergy Centre Odense University Hospital Odense Finland; ^84^ Department of Geriatrics Montpellier University Hospital, MUSE Montpellier France; ^85^ Department of Clinical and Surgical Sciences Fondazione Policlinico Universitario A Gemelli IRCCS Rome Italy; ^86^ National Heart and Lung Institute Royal Brompton Hospital & Imperial College London London UK; ^87^ Centre Hospitalier Valenciennes Valenciennes France; ^88^ Department of Internal Medicine (DiMI) University of Genoa Genova Italy; ^89^ IRCCS Ospedale Policlinico San Martino Genova Italy; ^90^ NOVA Medical School/Comprehensive Health Research Centre (CHRC) Lisbon Portugal; ^91^ Serviço de Imunoalergologia Hospital de Dona Estefânia Centro Hospitalar Universitário de Lisboa Central Lisbon Portugal; ^92^ Clinique des Bronches Allergie et Sommeil Hôpital Nord Marseille France; ^93^ Universidad Espíritu Santo Samborondón Ecuador; ^94^ Respiralab Research Group Guayaquil, Guayas Ecuador; ^95^ School of Medicine University CEU San Pablo Madrid Spain; ^96^ Department of Health Research Methods, Evidence, and Impact & Department of Medicine McMaster University Hamilton ON Canada; ^97^ Medical Faculty ENT Department Eskisehir Osmangazi University Eskisehir Turkey; ^98^ Department of Medical Sciences and Public Health and Unit of Allergy and Clinical Immunology University Hospital “Duilio Casula” University of Cagliari Cagliari Italy; ^99^ Allergy Division Chest Disease Department University Hospital of Strasbourg Strasbourg France; ^100^ Federation of Translational Medicine University of Strasbourg Strasbourg France; ^101^ VIM Suresnes, UMR 0892 Pôle des Maladies des Voies Respiratoires Hôpital Foch Université Paris‐Saclay Suresnes France; ^102^ Peercode BV Geldermalsen The Netherlands; ^103^ Department of Otorhinolaryngology, Head and Neck Surgery University Hospital of Crete Heraklion, Crete Greece; ^104^ Department of Chest Medicine Centre Hospitalier Universitaire UCL Namur Namur Belgique; ^105^ Université Catholique de Louvain Yvoir Belgium; ^106^ Ecole des Mines Alès France; ^107^ Allergist Reims France; ^108^ School of Health Sciences Catholic University of Salta Salta Argentina; ^109^ Berlin Institute of Health Berlin Germany; ^110^ Department of Pneumology Medical University of Gdańsk Gdańsk Poland; ^111^ Department of Clinical Immunology Wrocław Medical University Wrocław Poland; ^112^ ALL‐MED Medical Research Institute Wroclaw Poland; ^113^ Institute of Social Medicine, Epidemiology and Health Economics Charité ‐ Universitätsmedizin Berlin Berlin Germany; ^114^ Institute for Clinical Epidemiology and Biometry University of Wuerzburg Wuerzburg Germany; ^115^ State Institute of Health, Bavarian Health and Food Safety Authority Erlangen Germany; ^116^ Department of Clinical Science and Education Södersjukhuset Karolinska Institutet Stockholm Sweden; ^117^ Department of Pediatric Respiratory Diseases and Allergology Medical University of Warsaw Warsaw Poland; ^118^ Faculty of Health Sciences and CICS – UBI Health Sciences Research Centre University of Beira Interior Covilhã Portugal; ^119^ CIRFF Department of Pharmacy University of Naples Federico II Naples Italy; ^120^ CRI‐Clinical Research International‐Ltd Hamburg Germany; ^121^ Medical Communication Consultant Medscript Ltd, Dundalk Ireland and Wellington New Zealand; ^122^ Research Fellow OPC Cambridge UK; ^123^ Center of Allergy, Immunology and Respiratory Diseases Santa Fe Argentina; ^124^ Department of Allergy, Immunology and Respiratory Medicine Alfred Hospital and Central Clinical School Monash University Melbourne Victoria Australia; ^125^ Department of Otorhinolaryngology Charité‐Universitätsmedizin Berlin Berlin Germany; ^126^ Université Grenoble Alpes Laboratoire HP2 Grenoble France; ^127^ INSERM U1042 Villejuif France; ^128^ University of Liège Liège Belgium; ^129^ Conseil Général de l'Economie Ministère de l'Economie de l'Industrie et du Numérique Paris France; ^130^ Medical School University of Cyprus Nicosia Cyprus; ^131^ Department of Allergy Hospital La Paz Institute for Health Research (IdiPAZ) Madrid Spain; ^132^ Department of Otorhinolaryngology Amsterdam University Medical Centres, AMC Amsterdam the Netherlands; ^133^ Pneumologie AP‐HP Centre Université de Paris Cité Hôpital Cochin Paris France; ^134^ UMR 1016 Institut Cochin Paris France; ^135^ Pediatric Allergy and Clinical Immunology Hospital Espanol de Mexico Mexico City Mexico; ^136^ Asthma Reference Center ‐ School of Medicine of Santa Casa de Misericórdia of Vitória Vitoria, Espirito Santo Brazil; ^137^ National Heart and Lung Institute Imperial College London UK; ^138^ NIHR Imperial Biomedical Research Centre London UK; ^139^ Department of Respiratory Medicine and Tuberculosis University Hospital Brno Czech Republic; ^140^ Department of Medicine, Surgery and Dentistry “Scuola Medica Salernitana” University of Salerno Salerno Italy; ^141^ Imunoalergologia Centro Hospitalar Universitário de Coimbra Faculty of Medicine University of Coimbra Coimbra Portugal; ^142^ Department of General ORL H&NS Medical University of Graz ENT‐University Hospital Graz Graz Steiermark Austria; ^143^ Fihla, Finnish Lung Association Helsinki Finland; ^144^ University of Turku Turku Finland; ^145^ ASA ‐ Advanced Solutions Accelerator Jacou France; ^146^ Department of Medicine, Clinical Immunology and Allergy McMaster University Hamilton Ontario Canada

**Keywords:** asthma, digital, MASK‐air, mHealth, rhinitis

## Abstract

MASK‐air^®^, a validated mHealth app (Medical Device regulation Class IIa) has enabled large observational implementation studies in over 58,000 people with allergic rhinitis and/or asthma. It can help to address unmet patient needs in rhinitis and asthma care. MASK‐air^®^ is a Good Practice of DG Santé on digitally‐enabled, patient‐centred care. It is also a candidate Good Practice of OECD (Organisation for Economic Co‐operation and Development). MASK‐air^®^ data has enabled novel phenotype discovery and characterisation, as well as novel insights into the management of allergic rhinitis. MASK‐air^®^ data show that most rhinitis patients (i) are not adherent and do not follow guidelines, (ii) use as‐needed treatment, (iii) do not take medication when they are well, (iv) increase their treatment based on symptoms and (v) do not use the recommended treatment. The data also show that control (symptoms, work productivity, educational performance) is not always improved by medications. A combined symptom‐medication score (ARIA‐EAACI‐CSMS) has been validated for clinical practice and trials. The implications of the novel MASK‐air^®^ results should lead to change management in rhinitis and asthma.

## 1 INTRODUCTION

Most economies are struggling to deliver modern health care effectively. There is a need to support the transformation of the healthcare system into integrated care with organisational health literacy. Smart devices and Internet‐based applications (apps) are largely used in AR^1^ and may help to address some of the unmet needs in the real‐life assessment of patients' treatment choices and disease control. However, these new tools first of all need to be tested for privacy rules, acceptability, usability, validity and cost‐effectiveness. Second, they should be evaluated in the frame of the digital transformation of health, their impact on healthcare delivery and health outcomes so that mHealth tools may enable the digital transformation of health and care, empowering citizens and building a healthier society.^2^

AIRWAYS‐ICPs (Integrated care pathways for airway diseases) launched a collaboration to develop digitally‐enabled and multisectoral care pathways (ICPs).^3^ Initiated in 2013 under the frame of the European Innovation Partnership on Active and Healthy Ageing (EIP on AHA, DG Santé & DG Connect),^3^, ^4^ it was a GARD (Global Alliance against Chronic Respiratory Diseases, WHO) Research Demonstration Project.^5^, ^6^

Allergic rhinitis (AR), one of the most common chronic conditions globally, often co‐occurs with asthma and conjunctivitis (multimorbidity). It causes major burden and disability worldwide with substantial economic cost.^7^, ^8^ AR management is complex, as many possible interventions are available including allergen avoidance, pharmacotherapy and allergen‐specific immunotherapy (AIT).^9^, ^10^, ^11^, ^12^

Many evidence‐based guidelines for AR have improved its understanding and management.^12^, ^13^, ^14^, ^15^ They all propose long‐term continuous treatment for subjects with persistent symptoms. However, guidelines are mostly based on randomised controlled trials (RCTs), typically undertaken on highly‐selected samples of the population, often with limited/unclear generalisability to routine care contexts.^16^, ^17^, ^18^ Many patients are, however, dissatisfied with their treatment: despite high adherence to various treatment options, their symptoms remain poorly controlled. Moreover, adherence to treatment is usually poor in AR, even when using mHealth supporting tools.^19^

Large observational implementation studies are needed to triangulate RCTs and to better understand AR phenotypes and management. They reflect ‘real‐world’ everyday use and practice more closely than RCTs in terms of patient heterogeneity as well as the variety of medical interventions.^20^ Observational studies with direct patient data (often known as real‐world data) examine the possible effect of a treatment on subjects where the investigator has no control over the experiment and cannot randomise subject allocation.^21^ However, they provide clinically‐relevant information complementing RCT information. mHealth apps are a valuable source of direct patient data and offer new insights into chronic diseases.

As a tool for the implementation of AIRWAYS‐ICPs, MASK‐air^®^ (Mobile Airways Sentinel NetworK for airway diseases) is an mHealth app. It provides direct patient data and offers new insights into AR phenotypes and management in a patient‐centred approach in order to facilitate shared decision making.^22^ MASK‐air^®^ is a Good Practice of DG Santé for digitally‐enabled, patient‐centred care pathways.^23^ It helps to address certain unmet needs. MASK‐air has been reported in the JRC Scientific and Policy Reports on Strategic Intelligence Monitor on Personal Health Systems Phase 3 (SIMPHS3).^24^ This publication is a Science and Policy Report by the Joint Research Centre, the European Commission's in‐house science service. MASK‐air^®^ is one example of the WHO‐ITU (International Telecommunication Union) ‘Be He@lthy, Be Mobile’ handbook on how to implement mBreatheFreely for asthma and COPD.^25^

## 2 STRATEGIC OBJECTIVES OF MASK‐AIR^®^

### 2.1 Unmet needs

- The burden of AR (and multimorbidities) and unmet medical needs are unacceptable and require a novel approach to tackle them. Many patients with AR and/or asthma are:
  - uncontrolled
  - not satisfied by their treatment
- In all societies, the burden and cost of allergic and chronic respiratory diseases are increasing rapidly. Healthcare costs should be sustainable despite the increased prevalence of AR and the availability of new expensive treatments (e.g., biologics) for asthma multimorbidity.
- Most economies are struggling to deliver modern health care effectively, both in terms of
  - insufficient healthcare work force and
  - increasing costs.
- There are wide disparities within and between countries leading to underserved populations with increased burden.

### 2.2 Mission

- There is a need to support the transformation of the healthcare system into integrated care with organisational health literacy centred around the patient.
- mHealth apps and Internet‐based applications used in AR and asthma may help to address some of the unmet needs in the real‐life assessment of patients' treatment choices and disease control.
- However, these new tools need:
  - Firstly, to be tested for privacy rules, acceptability, usability and cost‐effectiveness.
  - Secondly, to be evaluated in the frame of the digital transformation of health to assess:
    - Their impact on healthcare delivery and health outcomes
    - so that mHealth tools may enable the digital transformation of health and care
    - empowering citizens
    - and building a healthier society.
- The ultimate goal is change management for AR and asthma multimorbidity.

### 2.3 Vision

To provide a novel cost‐effective strategy developing digitally‐enabled care pathways centred around the patient

- Using validated and user‐friendly mHealth tools.
- Based on patients' needs, beliefs, cultural differences and behaviour.
- To reduce the gaps between the patients and the physicians (to improve shared decision making).
- To provide next‐generation care pathways from the citizens to the specialist and the policy maker.
- To propose novel strategies with available treatments (and Value‐Added Medicine).
- To improve patients' health and well‐being and reduce indirect costs.
- This approach may need to define novel phenotypes with different medical needs.

The strategy should be deployed in the EU and globally (developed and developing countries) in order to reduce health and social inequalities within and between countries.

### 2.4 Objectives

**FIGURE 1:**
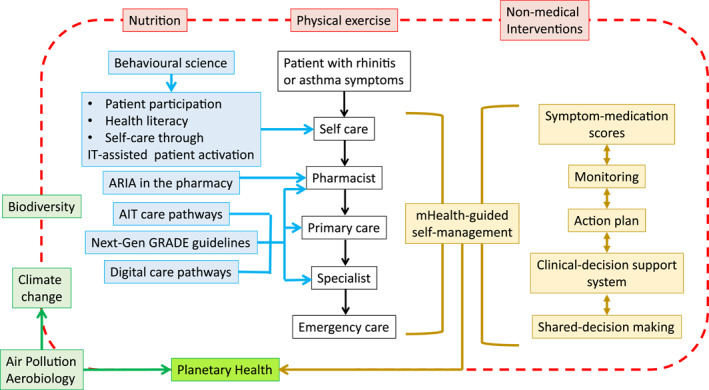
Overarching objectives of MASK‐air^®^.

The overarching objectives of MASK‐air^®^ are (i) to propose a multisectoral care pathway to transform healthcare systems in a cost‐effective manner in rhinitis and asthma using mHealth tools acceptable for the patient and the healthcare worker and (ii) to strengthen planetary health (Figure 1).

### 2.5 Strategic overview

The vision of MASK‐air^®^ has led to a strategic overview that was initiated by ARIA in 1999 (Table 1).

**TABLE 1:** Strategic overview (adapted from^22^)

Acronym	Name	Dates
WHO‐associated projects		
ARIA	Allergic Rhinitis and its Impact on Asthma	1999‐
WHO collaborating Centre on rhinitis and asthma		2004–2014
GARD	Global Alliance against Chronic Respiratory Diseases	2003‐
WHO‐ITU	‘Be He@lthy, Be Mobile’ handbook on asthma and COPD	2017
EU grants and projects		
GA^2^LEN	Global Allergy and Asthma European Network (FP6)	2004–
MeDALL	Mechanisms of the Development of Allergy (FP7)	2009–2014
EIP on AHA	European Innovation Partnership on Active and Healthy Ageing (DG Santé & CONNECT)	2012–2020
JCR Science and Policy Report	JRC Scientific and Policy Reports on Strategic Intelligence Monitor on Personal Health Systems Phase 3 (SIMPHS3)	2017
Twinning	Transfer of Innovation (DG Santé & CONNECT)	2017–2019
DHE twinning	Transfer of innovation in severe asthma (H2020)	2019–2020
POLLAR	Impact of Pollution on Asthma and Rhinitis (EIT Health)	2018–2019
Catalyse	Climate change (Horizon Europe)	2022–
Good Practice DG Santé on digital health (DG Santé)		2018
Candidate Best Practice OECD‐DG Santé		2023

Abbreviations: ARIA, Allergic Rhinitis and its Impact on Asthma; CARAT, Control of Allergic Rhinitis and Asthma Test; EAACI, European Academy of Allergy and Clinical Immunology; e‐CDSS, electronic clinical decision support system; GA^2^LEN, Global Allergy and Asthma European Network; GARD, Global Alliance against Chronic Respiratory Diseases; ITU, International Telecommunication Union; POLLAR, Impact of Pollution on Asthma and Rhinitis; WHO, World Health Organization.

## 3 mHEALTH APPS IN RHINITIS

Few apps addressing AR patients have been evaluated. This has made their selection difficult. We have introduced a new approach to market research for AR apps based on the automatic screening of the Apple App and Google Play stores.^26^ A JavaScript programme has been devised for automatic app screening and applied in a market assessment of allergic rhinitis self‐management apps. We searched the Google Play and Apple App stores of three countries (USA, UK, Australia) with the following search terms: hay fever, hayfever, asthma, rhinitis, allergic rhinitis. Apps were eligible if symptoms were evaluated. Three apps could be used in 2021, according to criteria required for the study, and two for the purposes of the Combined Symptom‐Medication Score (CSMS) (AllergyMonitor^27^, ^28^ and MASK‐air^®^).

## 4 MASK‐AIR^®^

### 4.1 Characteristics and geographical distribution

**FIGURE 2:**
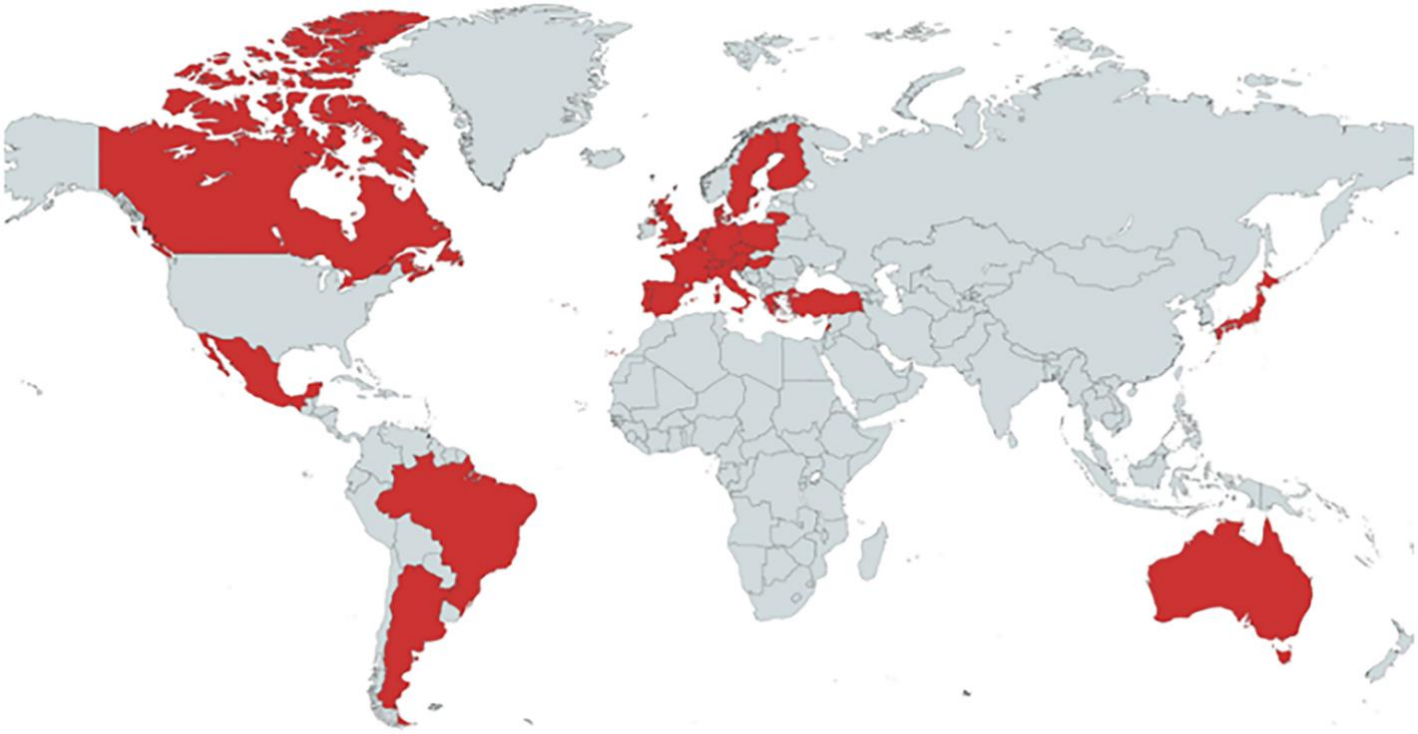
Geographical distribution of MASK‐air^®^.

MASK, the Phase 3 ARIA initiative, was developed from the MASK‐air^®^ app to a flexible e‐platform for allergic diseases and asthma. It is operational in 29 countries and 19 languages (Figure 2). Over 58,000 users have been registered.

MASK includes: (i) a freely available app (MASK‐air^®^, formerly the Allergy Diary, free on Android and iOS),^29^ (ii) tools to support healthcare professionals in shared decision making through an interoperable electronic decision support system (e‐CDSS),^30^ (iii) a web‐based interoperable questionnaire for physicians,^31^ (iv) a questionnaire on asthma and rhinitis (CARAT: Control of Allergic Rhinitis and Asthma Test) for screening allergic diseases and assessing their control^32^, ^33^ and (v) a sentinel network for air quality (air pollution) and pollen seasons.^34^ The MASK‐air^®^ app is centred around the patient.^35^

### 4.2 General Data Protection Regulation and Medical Device regulation

MASK‐air^®^ (formerly the Allergy Diary) was CE1 registered. It has now also been registered as MRD class IIa ((MDD) REGULATION (EU) 2017/745) in order to be included in electronic files (https://eur‐lex.europa.eu/legal‐content/EN/TXT/PDF/?uri=CELEX:32017R0745).

The download and usage of this App are free of charge and there are no advertisements. It falls under French jurisdiction.

It follows the General Data Protection Regulation (GDPR) which regulates the processing of personal data in the European Union (EU).^36^ Geolocation also follows the GDPR.^37^

### 4.3 Maturity level

The Technology Readiness Level (TRL)^38^ has been assessed (Table 2).

**TABLE 2:** Maturity level of MASK‐air

Rhinitis platform	TRL	References	Asthma platform	TRL	References
App for rhinitis and multimorbidity (MASK‐air^®^): available in 28 countries, 17 languages, >50,000 users	9	^35^, ^39^, ^40^	Adaptation of the MASK‐air app for SA developed and tested by the DHE SA‐TWINNING	8	
PROMs for rhinitis	9		PROMS for asthma	9	^41^
CARAT questionnaire for screening and control of rhinitis and asthma, 20 countries	9	^32^, ^42^, ^43^, ^44^	The same questionnaire will be used	9	^32^, ^42^, ^43^
e‐physician questionnaire for rhinitis (available on the MASK‐air website) deployed in 28 countries and 20 languages	9	^31^	Adaptation of the MASK questionnaire for SA developed by the DHE SA‐TWINNING	6	
Electronic clinical decision support system in English for rhinitis	8	^30^			
Embedding air quality (outdoor air pollution) and pollen data in MASK‐air^®^ (POLLAR)	9	^45^	Alerts for air pollution and pollens predicting asthma exacerbations	5	
	NA		Alerts for rhinovirus predicting asthma exacerbations	4	^46^, ^47^
EAACI‐ARIACARE‐digital network (28 countries, 20 languages)	9		The same network will be used	9	
Symptom‐medication score for rhinitis	9	^48^	Daily control‐medication score for asthma	5	
	NA		Sensors for pulmonary function	5	
Embedding artificial intelligence in MASK‐air^®^	2		Embedding artificial intelligence in MASK‐air^®^	2	
GDPR for the app	9	^37^, ^49^	GDPR for the app	8	^37^, ^49^

### 4.4 Validation

COSMIN guidelines^50^ were assessed for the VAS scales used in MASK‐air^®^ (Table 3). There was an excellent internal consistency (Cronbach's test >0.84, test‐retest >0.7), reliability (>0.9) and acceptability. In addition, the VASs had a good sensitivity when users (*n* = 521) answered them twice in <3 h. In a second study,^51^ intra‐rater reliability was tested (intraclass correlation coefficients, ICCs) and ranged from 0.870 to 0.937. Test‐retest reliability in clinically‐stable users ranged from 0.604 to 0.878. Responsiveness (Cohen's effect size and standardised response mean) was assessed in users with two consecutive measurements of EQ‐5D VAS or ‘VAS Work’ indicating clinical change. Moderate/large effect sizes were observed (highest responsiveness for VAS global allergy and lowest responsiveness for VAS sleep). The quality of data was checked in MASK‐air^®^ ^52^ using insufficient effort responding (IER)^52^ to assess the intra‐individual response variability (IRV) index.^52^ The independency of VAS questions was assessed using the Bland and Altman regression analysis.^75^ The analysis showed that all VAS measurements were independent.^59^

**TABLE 3:** Methodologic validation and achievements of MASK‐air^®^

	Study name	Ref	Type of study	N users	N days	N countries
Methodology						
1	COSMIN guidelines	^50^	Obs, CS‐L	2497	14,612*	15
2	Test‐retest, intra‐class coefficient	^51^	Obs, CS‐L	17,780	317,176	25
3	Quality of data (intra‐individual response variability)	^52^	Obs, CS	14,189	205,904	23
4	Independence of data	^51^	Obs, CS	1136	5889	18
5	EQ‐5D	^53^ ^,^ ^54^	Obs, CS	1288	NA	18
6	WPAI‐AS	^53^ ^,^ ^54^	Obs, CS	1288	NA	18
7	CARAT	^44^	Obs, CS	1086	2042	22
8	CHRODIS guidelines	^55^	Obs, CS	NA	NA	NA
Major achievements						
9	Pilot study of mobile phone technology in AR in European countries. The MASK‐rhinitis study	^56^	Obs, CS	3260	NA	20
10	Adherence to treatment of AR using mobile technology	^57^	Obs, CS	6949	NA	21
11	Treatment of AR using mobile technology with real‐world data: The MASK observational pilot study	^58^	Obs, CS	2871	39,634	
12	Work productivity in rhinitis using cell phones: The MASK pilot study	^59^	Obs, CS	1136	5818	21
13	Behaviour of MASK‐air^®^ users	^60^	Obs, CS	13,122	222,024	27
14	The Work Productivity and Activity Impairment Allergic Specific (WPAI‐AS) Questionnaire Using Mobile Technology: The MASK Study	^61^	Obs, CS	1288	1288	18
15	Correlation between work impairment, scores of rhinitis severity and asthma using the MASK‐air^®^ App	^48^	Obs, CS	14,189	205,904	23
16	Mobile technology offers novel insights into the control and treatment of AR. The MASK study	^40^	Obs, CS	9122	112,054	23
17	Treatment of AR during and outside the pollen season using mobile technology. A MASK study	^62^	Obs, CS	9035	70,286	Europe 18
18	Validation of the MASK‐air App for assessment of AR	^63^	RCT	267	7500	Spain
19	Effect of nasal irrigation on AR control in children; complementarity between CARAT and MASK outcomes	^64^	RCT	76	NA	Greece
20	Implementation of the MASK‐air^®^ app for rhinitis and asthma in old age adults. MASK@Puglia	^65^	Obs, CS	174	NA	Italy
21	Mobile health app for monitoring AR and asthma in real life in Lithuanian MASK‐air users	^66^	Obs, L	149	NA	Lithuania
22	Development and validation of combined symptom‐medication scores for AR (ARIA‐EAACI CSMS)	^67^	Obs, CS	17,780	317,176	25
23	Differences in behavioural patterns in AR medication in Europe: A study using MASK‐air^®^ real‐world data	^60^	Obs, CS	13,122	222,024	Europe 18
24	Comparison of rhinitis treatments using MASK‐air^®^ data considering the Minimal Important Difference	^68^	Obs, CS	10,860	269,837	28
25	Academic productivity in AR: A MASK‐air^®^ direct data cross‐sectional study	^69^	Obs, CS	1970	13,454	28
26	Asthma PROMs in severe asthma	^41^	Obs, CS	86	2349	12
27	Longitudinal severe asthma pilot study	^70^	Obs, CS	13	1250	Italy
28	Clusters of asthma and rhinitis	Submitted	Obs, CS	8075	297,169	26
29	Longitudinal assessment of MASK‐air^®^ data in AR using cluster analysis (16,000 weeks)	Submitted	Obs, L	2590	113,239	25
30	Effect of AIT in the MASK‐air^®^ study: proof‐of‐concept analysis	^71^	Obs, CS	17,780	317,176	25
31	Allergen AIT in MASK‐air users in real‐life: results of a Bayesian mixed‐effects model	^72^	Obs, CS	1093	42,756	25
32	Daily improvement of allergy control by sublingual AIT: A MASK‐air^®^ cross‐sectional study	Submitted	Obs, CS	217	4726	14
33	POLLAR	^73^	Obs, CS	3323	36,440	15
34	Comparison of anti‐histamine reporting by MASK‐air, Google Trends and sales in Europe	^74^	Obs, CS			
35	Electronic daily symptom‐medication score in asthma (eDASTHMA)	Submitted	Obs, CS	1662	135,635	27
36	Cut‐off values of PROMs	Submitted	Obs, CS	23,201	395,223	27
37	Treatment of asthma using mHealth real‐world data: The MASK‐air observational study		Obs, CS	3229	70,270	27
38	Disentangling rhinitis and asthma using MASK‐air	Submitted	Obs, CS + L	3797	256,839	27
39	MASK‐air in old age adults	Submitted	Obs, CS	19,888	349,045	27
40	Adherence to ICS/LABAs in asthma		Obs, CS			

Abbreviations: CS, cross‐sectional; L, longitudinal; NA, not available; Obs, Observational; RCT, randomised controlled trial.

### 4.5 Achievements

The overall results concerning methodological validation and achievements are presented in Table 3.

### 4.6 Patient‐reported outcome measures (PROMs) in rhinitis

#### 4.6.1 Visual analogue scales

**FIGURE 3:**
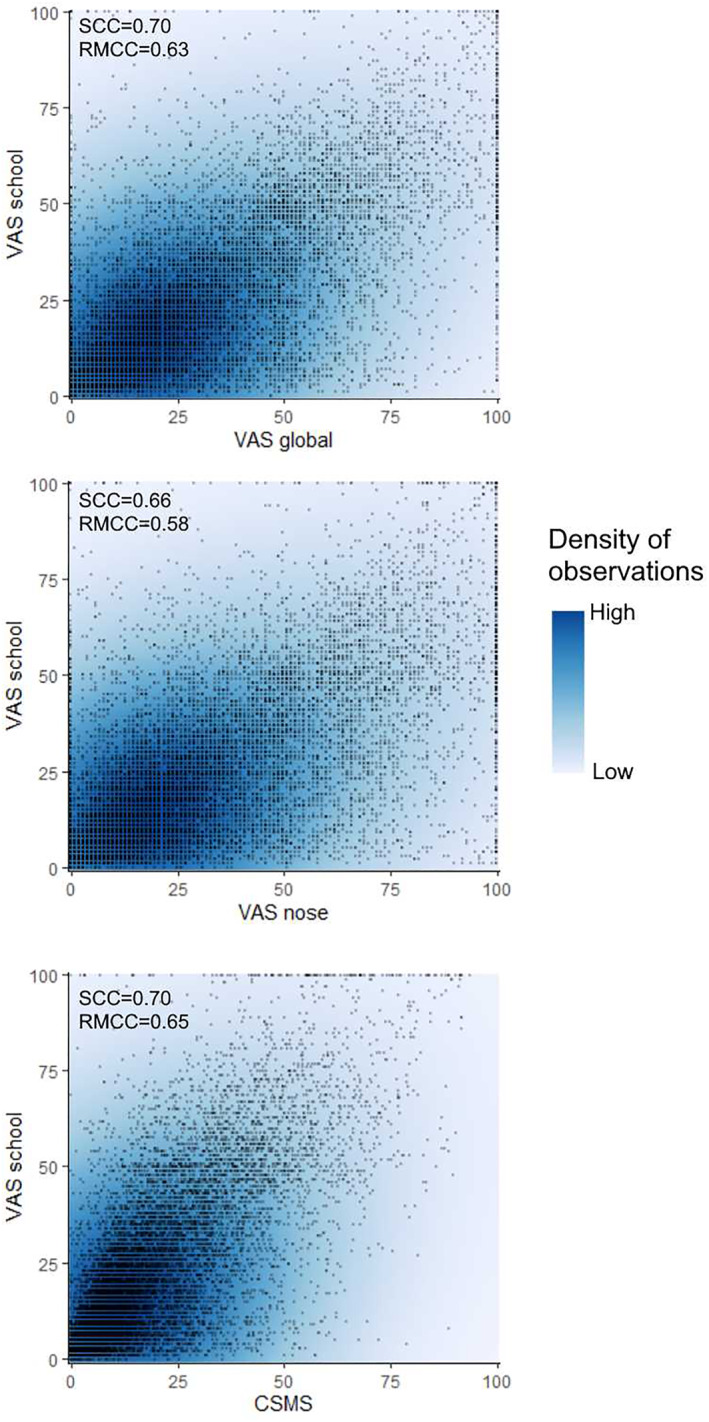
Correlations between some of the rhinitis PROMs (unpublished).

Even though there is an independence of data in MASK‐air, all PROMs are highly correlated (Figure 3).^40^, ^58^, ^59^, ^61^, ^62^, ^67^ It is unlikely that the results can be explained by a low quality of data arising from repeated VAS measures.^48^ PROMs (VAS global, nose, eye, work, sleep and asthma^41^) can be used in clinical trials, observational studies and clinical practice. PROMs for asthma have also been evaluated.^41^, ^44^

#### 4.6.2 Cut‐off values for PROMs

Patient classification into groups based on the value of a PROM may help to apply different care or procedures. A continuous variable may have clinical significance concerning the outcome, but its effects may be non‐linear or non‐monotonic. Based on a study on VAS assessment and according to the ARIA classification^76^ and the ICF (International Classification of Functioning) grading,^77^ we proposed arbitrary cut‐offs in MASK‐air. However, cut‐offs can be calculated. There are two statistical approaches for determining a cut‐off value: PROM‐oriented (percentiles) and outcome‐oriented (VAS work and EQ‐5D). Moreover, it is important to determine the ‘no symptom’ level (Sousa‐Pinto, submitted) (Table 4).

**TABLE 4:** MASK‐air cut‐off levels

					Arbitrary^76^	Outcome‐oriented	
						Global, nose, asthma	Eye
ICF^77^	Full problem	0%–4%	MASK‐air VAS (0–100)^23^	Full control	0	0	0
	Mild problem	5%–24%		Control	1–19	1–19	1–12
	Moderate problem	25%–49%		Partial control	20–50	20–36	13–39
	Severe problem	50%–95%		No control	≥50	≥36	≥36
	Complete problem	96%–100%					

### 4.7 Electronic daily combined symptom‐medication score (ARIA‐EAACI CSMS)

Validated combined symptom‐medication scores (CSMSs) are needed to investigate the effects of AR treatments.

The gold standard for the assessment of a CSMS requires a tool that does not include symptoms or medications and, if possible, has an economic impact. Such tools include, among other end points, work productivity and quality‐of‐life.

MASK‐air^®^ data have assessed the concurrent validity, test‐retest reliability and responsiveness of one hypothesis‐driven CSMS (modified CSMS: mCSMS),^48^ one mixed hypothesis‐ and data‐driven score (mixed score) and several data‐driven CSMSs generated by cluster analysis and regression models or factor analysis. These CSMSs were compared with scales measuring (i) the impact of rhinitis on work productivity (visual analogue scale [VAS] work of MASK‐air^®^ and Work Productivity and Activity Impairment: Allergy Specific [WPAI‐AS]), (ii) quality‐of‐life (EQ‐5D VAS) and (iii) control of allergic diseases (CARAT).^67^

The following scores were defined and tested (Table 5) and then validated in different countries.

**TABLE 5:** CSMS validation

A hypothesis‐driven score (m‐CSMS) built without knowing real‐life data moderately correlated with the 4 outcomes (Spearman rank correlation with VAS work: *p* = 0.61, *N* = 120,959). A mixed data‐ and hypothesis‐driven score (MIXED score) built based on real‐life data obtained in MASK highly correlated with the 4 outcomes (Spearman rank correlation with VAS work: *p* = 0.81, *N* = 118,275). Six data‐driven cluster‐based CSMSs built from clusters based on VAS work and EQ5D (3 CSMS) and CARAT and WPAI‐AS (3 CSMS) highly correlated with the 4 outcomes (Spearman rank correlation with VAS work: *p* = 0.73–0.83, *N* = 57,527–123,123). One regression‐based MIXED‐CSMS built from MASK‐air data correlated with the 4 outcomes (Spearman rank correlation with VAS work: *p* = 0.81, *N* = 94,399–128,123). A factorial analysis method (1 score) had a poor correlation with the 4 outcomes (Spearman rank correlation with VAS work: *p* = 0.42, *N* = 59,378).

**FIGURE 4:**
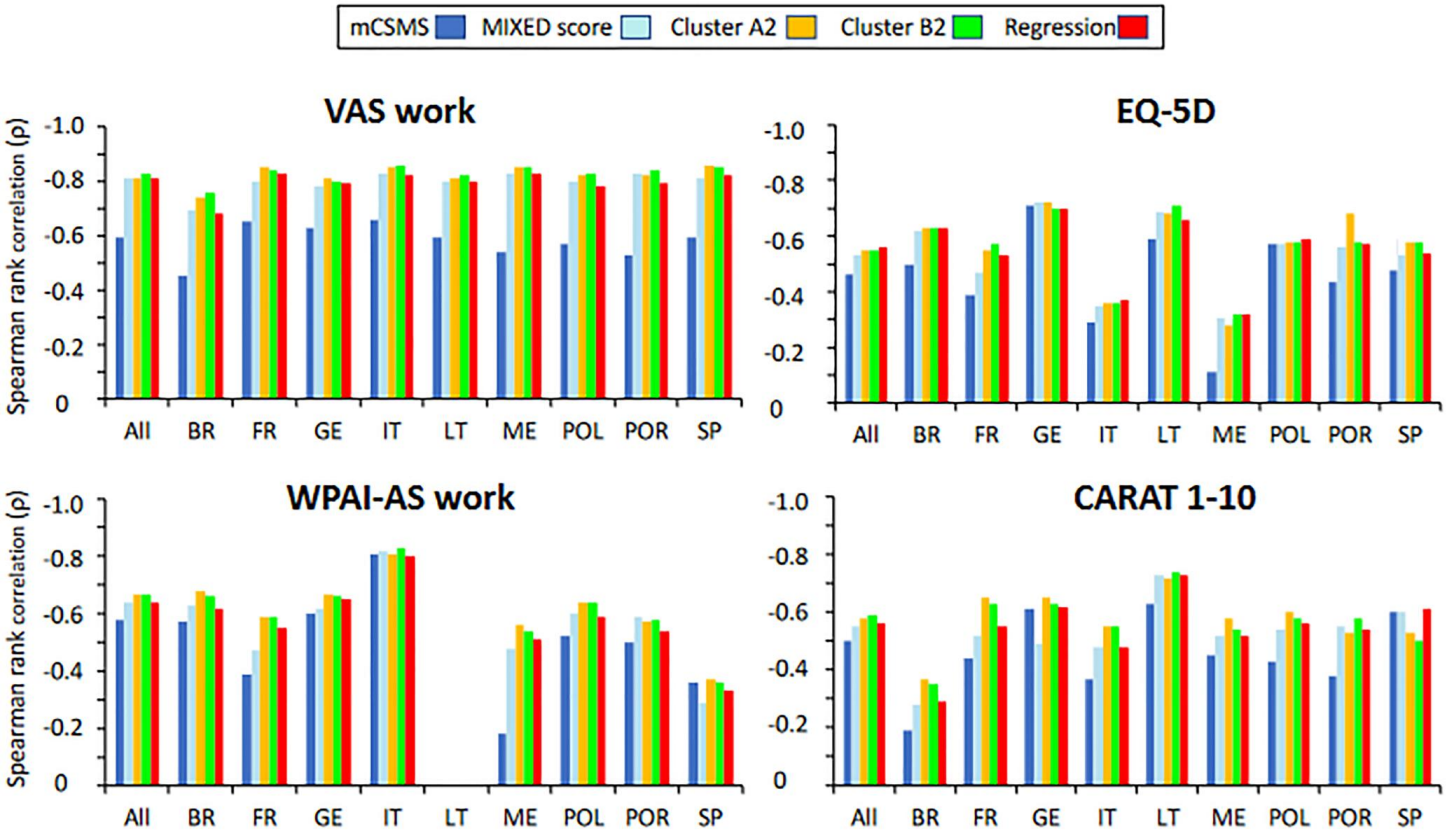
Reproducibility of CSMSs in different countries (from^67^).

There was a very high reproducibility of CSMSs in the 9 countries where there were enough data to provide statistical analyses (Figure 4).

### 4.8 Transfer of innovation

A transfer of innovation was carried out. The rhinitis Twinning made it possible to build an Interoperable platform with MASK. 25 Reference Sites of the European Innovation Partnership on Active and Healthy Ageing participated as well as Argentina, Australia, Brazil, Canada and Mexico.^78^, ^79^, ^80^, ^81^

## 5 OVERALL LIMITATIONS AND STRENGTHS OF MASK‐AIR^®^ STUDIES

### 5.1 Limitations

#### 5.1.1 Those of mHealth observational studies

- There are potential measurement biases when using apps since the information collected is usually restricted and less complete than when using more detailed paper or web‐based questionnaires.
- App users may be a selected subset and are not fully representative of all AR patients in the general population. Higher education or specific age ranges might apply.
- Precise patient characterisation is impossible via an app used in real life. However, every study in MASK‐air has produced highly‐consistent results with a clear perspective.
- The diagnosis of rhinitis, asthma and/or conjunctivitis is not confirmed by a physician. Users self‐report symptoms but the baseline questionnaire on rhinitis and conjunctivitis as well as CARAT for rhinitis and asthma help the diagnosis.
- Information biases associated with the underreporting of medication use are possible.
- There is an unsupervised input of data.
- Observational studies can only be hypothesis‐generating and findings should be confirmed by proper studies.

#### 5.1.2 Most studies reported in MASK‐air^®^ are cross‐sectional

- In MASK‐air, we used a cross‐sectional approach, taking days as the unit of analysis instead of patients (although patients were used to cluster reporting days). This approach has been applied in many studies^39^, ^48^, ^58^, ^62^, ^73^ and may have brought new information.
- Cross‐sectional studies may provide different results if different timeframes are chosen. However, we ruled out this bias.^62^
- Cross‐sectional studies cannot provide definite information about temporal relationships, let alone cause‐and‐effect relationships (causal inference). By contrast, longitudinal studies can establish sequences of events and allow the establishment of links or associations between variables.
- A longitudinal study with MASK‐air data has shown that longitudinal results are consistent with previous cross‐sectional data (submitted).

Although analyses have been carried out in over 28,000 users in 27 countries, a replication study is not available.

### 5.2 Strengths

**FIGURE 5:**
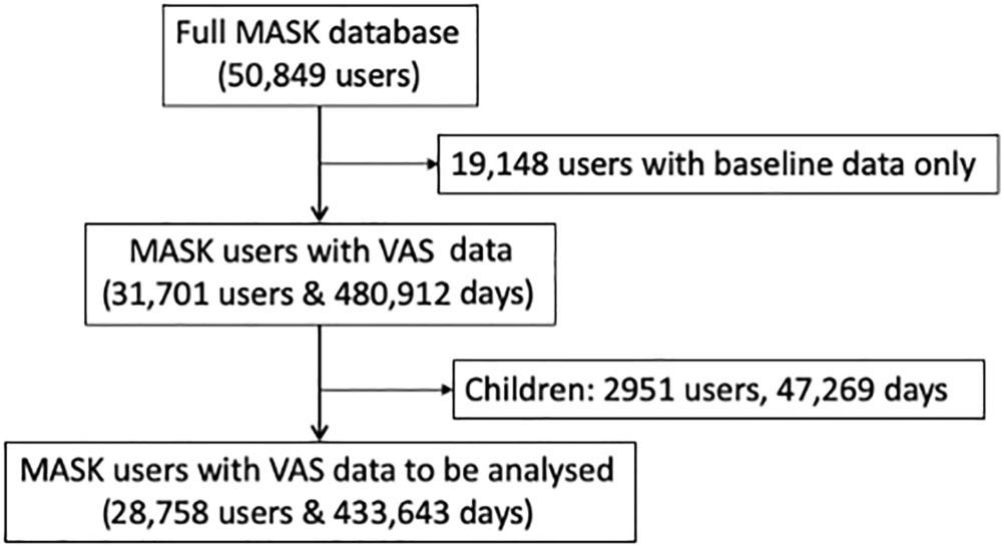
Repartition of MASK‐air^®^ users (December 2021).

- Overall, MASK‐air^®^ has several strengths: Low cost, quickly available data, large sample from 29 countries, reproducibility of results between countries (generalisability), individual reports to guide management and shared decision making, patient and physician education for a proactive role, assessment of new interventions or consequences of naturally occurring phenomena and climate change.
- The MASK‐air and ARIACARE networks.
- The MASK‐air^®^ app is available in 29 countries (20 languages) and is inter‐operable with a web‐based physician's questionnaire^82^ and an e‐CDSS for AR.^30^
- It currently (September 2022) includes over 58,000 users and around 600,000 days (Figure 5).
- The MASK‐air database does not have any missing values due to the structure of the app.
- It is an MDR Class IIa.
- It is fully validated.
- It includes pollen data (daily and predictive) based on the patients' geolocation (and pollution, 2022).^34^, ^73^
- It is a Good Practice of DG Santé for digitally‐enabled, patient‐centred care and a candidate Good Practice of OECD. In 2019, the European Commission embarked on a project with the OECD (Organisation for Economic Co‐operation and Development) entitled ‘The Best Practice Project’. This project was designed to produce evidence to assist policy‐makers of Member States in the selection, implementation and evaluation of best practice interventions, targeting non‐communicable diseases and digitally‐enabled integrated care. As part of this project, the OECD develops a range of analyses based on the case studies of candidate best practices. MASK has been chosen by DG Santé as a candidate best practice, and the document is under review for approval in 2022. Each case study focusses on:
  - An analysis of the intervention against a range of criteria, including effectiveness and efficiency.
  - A range of policy options to help policy‐makers enhance the performance of the intervention.
  - An assessment of how transferable the intervention is to other Member States.
- The rhinitis assessment is nearing completion in over 20,000 users (current paper) and the asthma assessment has been initiated in over 8000 users.
- In asthma, all categories of patients are included and the database can be used to compare asthmatics of different severity grade.

### 5.3 Economic evaluation

The economic evaluation is currently being assessed and several MASK‐air^®^ tools can be compared. They include the cost of medications effectively used, the cost of absenteeism and presenteeism (VAS Work, WPAI‐AS), costs of health resource utilisation (EQ‐5D VAS) and potential benefits of expensive treatments such as allergen immunotherapy and biologics. Combining the results of these tools, a monetary value will be ascribed to the CSMS.

## 6 NOVEL PHENOTYPES OF ALLERGIC DISEASES

### 6.1 Discovery of the ‘extreme allergy‐asthma phenotype’

Multimorbidity in allergic airway diseases was well known, but no data existed on the daily dynamics of symptoms and their impact on work.

**FIGURE 6:**
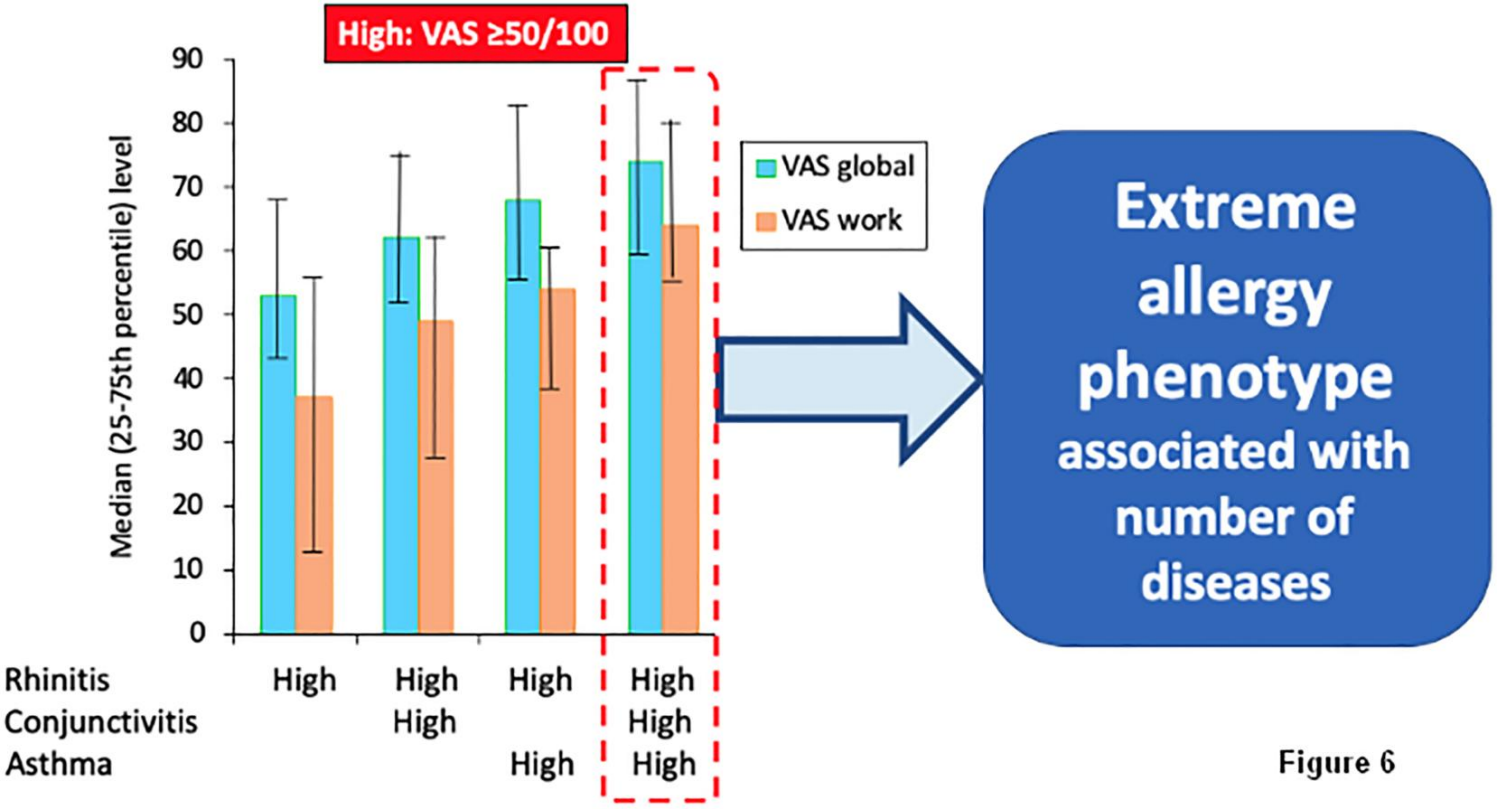
The extreme allergy phenotype (from^39^).

A cross‐sectional observational study was performed on 4210 users, 32,585 days and 19 countries.^39^ Five VASs assessed the daily burden of the disease (global, nose, eyes, asthma and work). VAS levels <20/100 were categorised as ‘Low’ burden and VAS levels ≥50/100 as ‘High’ burden. Eight hypothesis‐driven patterns were defined. A novel Rhinitis High—Asthma High—Conjunctivitis High pattern was identified in 2.9% of days. They had the greatest impact on uncontrolled VAS global measured and impaired work productivity (Figure 6).

**Limitations of the study:** Overall limitations.

### 6.2 Confirmation by canonical epidemiologic and genetic studies

mHealth apps are only tools that generate hypotheses. They therefore need to be confirmed in classical epidemiologic studies. Differences between AR alone and AR associated with conjunctivitis were already known.^83^, ^84^, ^85^ However, new studies carried out using MASK‐air^®^ data have shown that (i) ocular symptoms are more common in polysensitised patients whether or not they have asthma,^86^ (ii) ocular symptoms are associated with the severity of nasal symptoms,^87^, ^88^ (iii) it is important to consider ocular symptoms in severe asthma^87^ and (iv) the severity of allergic diseases increases with the number of allergic multimorbidities.^89^ A genomic approach showed differences between diseases alone and multimorbidity.^90^

### 6.3 Confirmation in MASK‐air studies

#### 6.3.1 ‘Asthma’ key words (cross‐sectional study)

**FIGURE 7:**
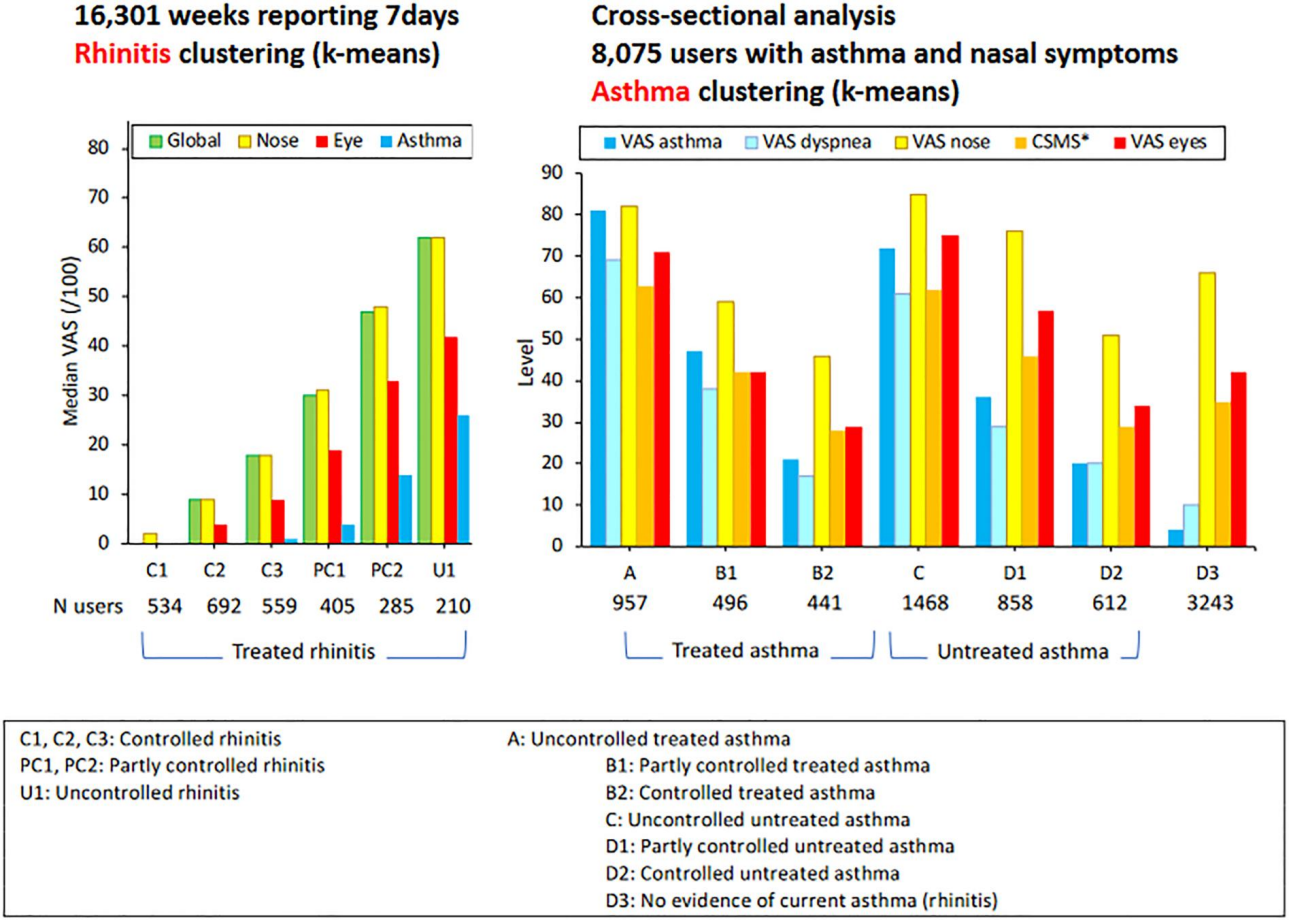
Phenotypes of rhinitis clusters in a longitudinal study and asthma in a cross‐sectional study.

In a **cross‐sectional asthma cluster analysis**, an extreme asthma phenotype was found independently of treatment. This phenotype was associated with high rhinitis, high conjunctivitis and high CSMS. It can be separated into 2 groups (patients with and without a treatment for asthma). Patients with treated uncontrolled severe asthma have more uncontrolled rhinitis than untreated ones (Figure 7).

#### 6.3.2 ‘Rhinitis’ key words (longitudinal study)

In a **longitudinal rhinitis cluster analysis**, two extreme allergy phenotypes were identified (Figure 6). One was associated with uncontrolled asthma (U1) and a second one with controlled asthma (U2). The first phenotype (U1) was unchanged when several weeks were analysed, whereas the second one (U2) was unstable and often associated with uncontrolled asthma when several weeks were analysed. This study confirms that the ‘extreme’ allergy phenotype is found in patients and that the three diseases are associated with a significant impact on work productivity (Figure 7).

**Limitations of the studies**: Only patients with asthma and nasal symptoms were studied.

#### 6.3.3 Confirmation of the distinct rhinitis and rhinitis + asthma phenotypes

A study compared reported symptoms and medication use in rhinitis versus A + R using direct patient data from the MASK‐air^®^ mHealth app (3797 patients and 256,839 days). Patients with rhinitis and (A + R) required more rhinitis medications and had more severe VAS levels for ocular symptoms and work than those with only rhinitis. The allergy‐CSMS was also higher in A + R patients than in rhinitis patients. Robust results were obtained when assessing 12 individual countries showing generalisability or in a sub‐analysis of 282 patients enroled by physicians. Moreover, in 14,409 complete weeks, the distribution of uncontrolled rhinitis weeks increased from rhinitis to R + possible asthma and R + probable asthma.

## 7 INTERPRETATION OF DIRECT PATIENT DATA FOR THE PHARMACOLOGIC TREATMENT OF ALLERGIC RHINITIS

### 7.1 Adherence to rhinitis treatment is poor

An observational cross‐sectional study has assessed the adherence to treatment in AR patients using MASK‐air.^57^ Secondary adherence was assessed by using the modified Medication Possession Ratio (MPR) and the Proportion of Days Covered (PDC) approach. 1887 users reported ≥7 days of VAS data. 11.3% of users were adherent (MPR ≥70% and PDC ≤1.25), 4.2% were partly adherent (MPR ≥70% and PDC = 1.50) and 14.6% were switchers. When physicians are allergic, they behave like patients,^91^ which suggests the need for behavioural science to improve control.

**Limitations of the study:** We only considered the users who reported over 6 days of MASK‐air use. We did not analyse the type of treatment due to its great variability. We did not include a questionnaire on medication adherence.

### 7.2 Patients treat themselves when they are not well

**FIGURE 8:**
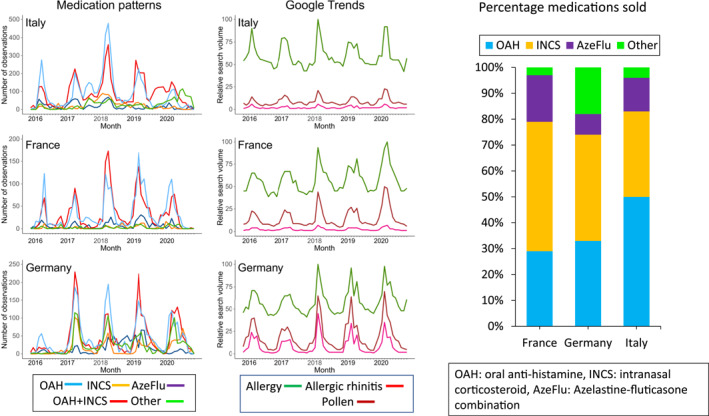
Behavioural patterns of medication usage in MASK‐air^®^.

Medication use peaked during the pollen season in all of the investigated European countries^61^ (Figure 8) whereas cultural behaviour ‐ assessed using Google Trends^92^ ‐ differed. Oral antihistamines (OAH) were the most common medications reported in monotherapy and comedication. This is against guideline recommendations and does not accord with the dispensing of medications (OTC and prescribed) in the pharmacy.^92^

**Limitations of the study:** European MASK‐air^®^ users are not fully representative of European patients with AR, posing generalisability concerns.

### 7.3 Many patients use OTC medications and self‐medicate

**FIGURE 9:**
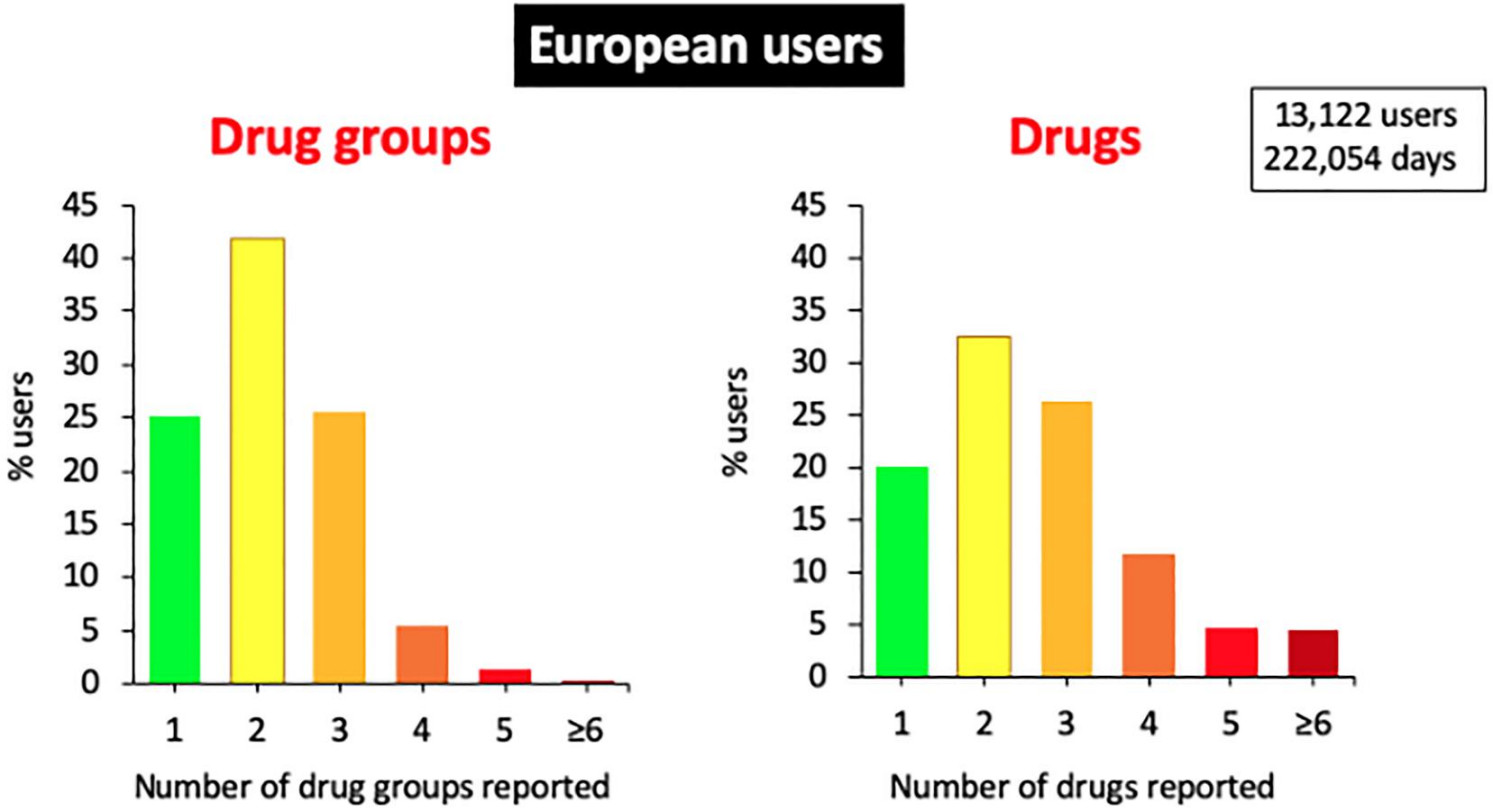
Number of medications and medication groups reported in Europe.

A large number of patients use OTC medications and self‐medicate. In Europe, users reported an annual average of 2.7 drugs, with 80% reporting two or more (Figure 9).

### 7.4 Switching of treatments is common (longitudinal analysis)

**FIGURE 10:**
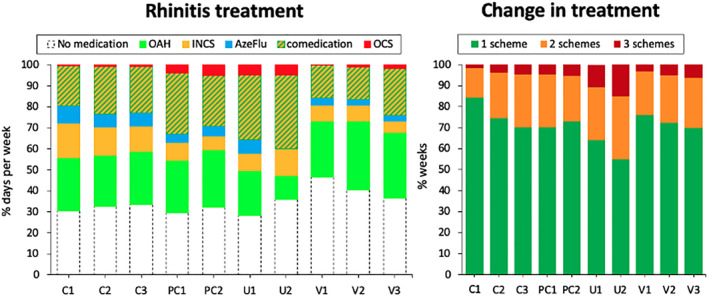
Rhinitis treatment and change of treatment in users reporting app data 7 days a week.

A longitudinal analysis of MASK‐air^®^ data assessed 16,177 weeks of patients answering to the daily monitoring questionnaire on all 7 days (Figure 10). K‐means cluster analysis methods were applied in order to group weeks according to their daily AR control. Ten clusters of users receiving a treatment were identified: 3 controlled (C1‐C3), 2 partly‐controlled (PC1‐PC2), 2 uncontrolled (U1‐U2) and 3 with variable control (V1‐V3) (Figure 9). In these users, no medication was reported in 30%–40% of days. Moreover, comedication (INCS/AzeFlu + Other) was reported in 19%–32%. Except for the most severe cluster (U2), OAH were reported in around 20%–25% of days. Change of treatment within a week was reported in 15% (C1) to 42% (U2) of days, and change of two treatments from 2% to 12% of days.

### 7.5 Increasing the number of medications is associated with impairment of control

**FIGURE 11:**
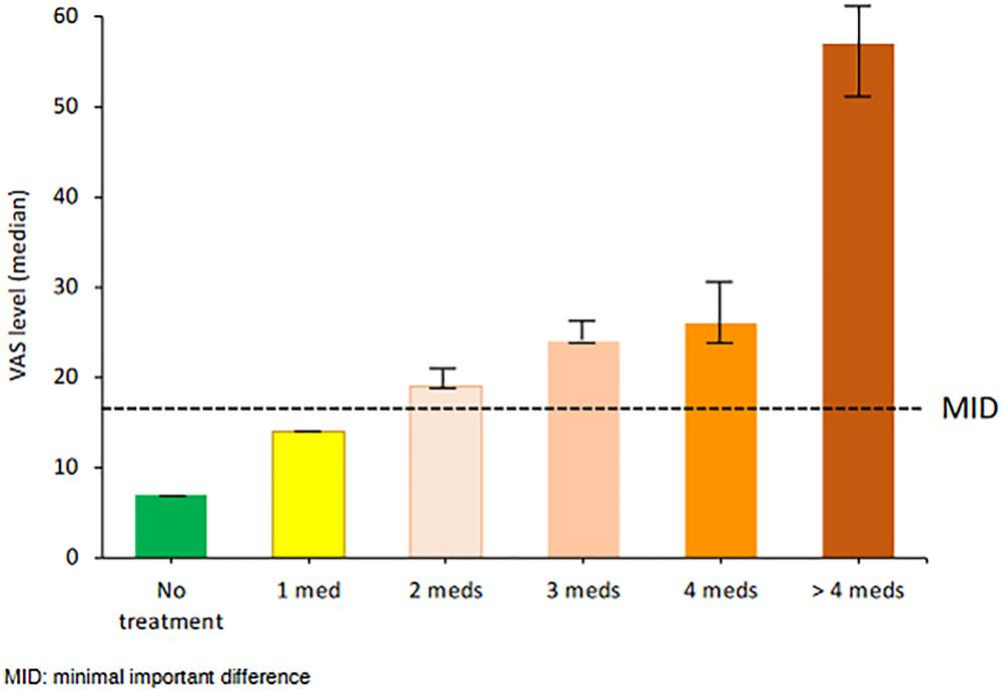
Daily VAS for global allergy symptoms depending on the number of medications reported.

Control worsens when the number of medications increases (Figure 11). This finding does not accord with guidelines proposing that medications should be stepped‐up to achieve control. However, this does accord with the concept of SCUAD (Severe Chronic Upper Airway Disease)^93^ and the severity of certain chronic diseases associated with multiple medications.

**Limitations of the study:** European MASK‐air^®^ users are not fully representative of European patients with AR, posing generalisability concerns.

### 7.6 Comedication is associated with impaired control by comparison to monotherapy

**FIGURE 12:**
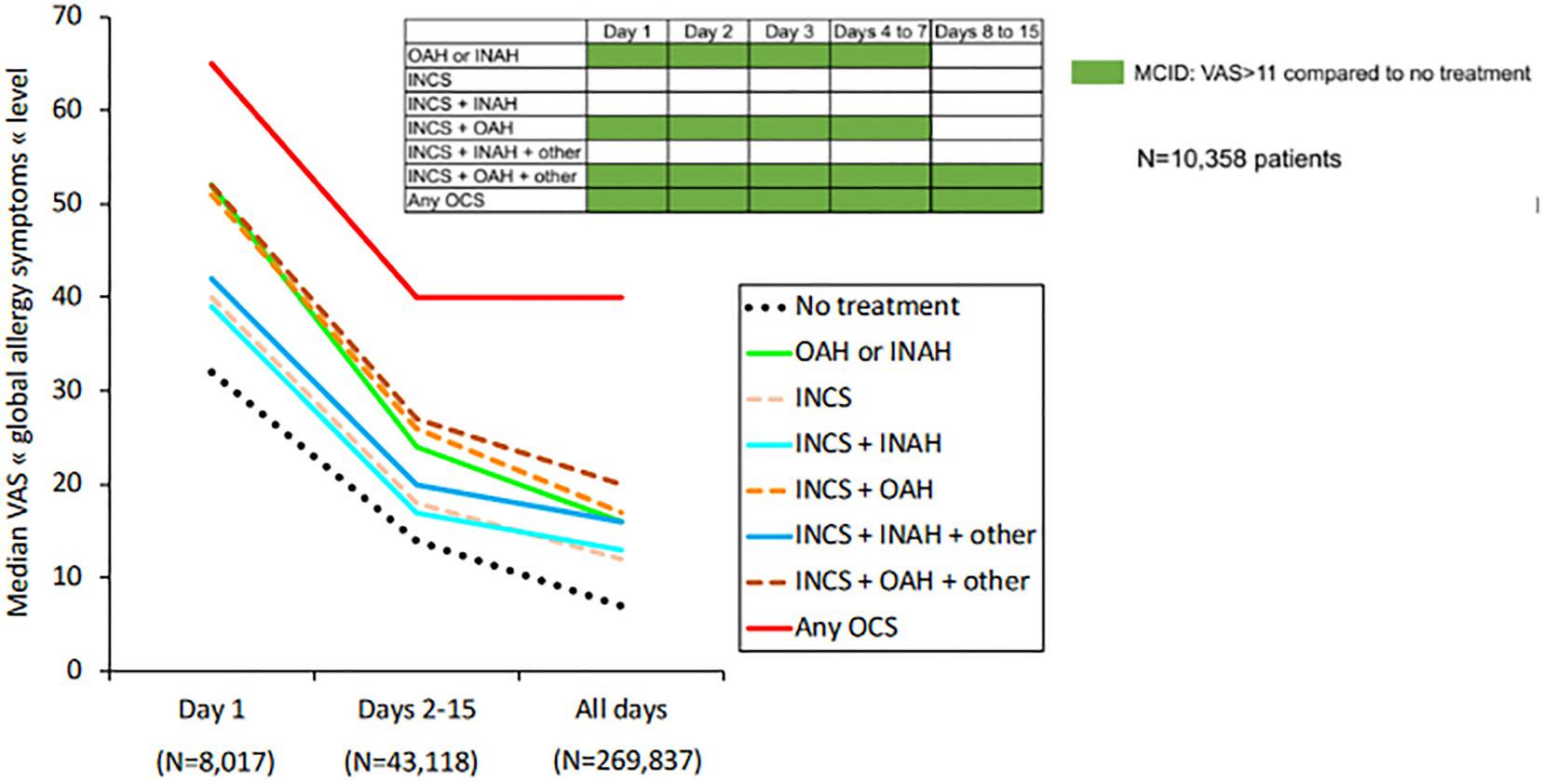
Daily VAS global allergy symptoms depending on the day of the treatment reporting.^68^

In several papers, the same results were observed: comedication is associated with worse control by comparison to monotherapy with INCS or Aze‐Flu.^40^, ^58^, ^62^, ^68^ Days with the best control were those with no medication. The same results were observed during and outside the pollen season. In all four studies, the trends between medications were similar from the first day of reporting to long‐term reporting. However, the levels of VAS global allergy symptoms decreased largely with time in treated and untreated days (Figure 12).

**Limitations of the study:** Overall limitations.

## 8 TOWARDS A NEW VISION OF AIT

### 8.1 AIT is effective using direct patient data

Evidence regarding AIT efficacy on AR has been provided mostly by RCTs. A pilot study showed that AIT was able to improve symptoms and work productivity (Table 6). This study suggested an additive effect of AIT over medications.^71^

**TABLE 6:** Impact of AIT on real‐world data using MASK‐air^®^

Medication scheme	N observations (%)		Symptoms VAS—median [95%CI] (IQR)		*p* value
	AIT	No AIT	AIT	No AIT	
All observations	36,229 (11.4)	280,947 (88.6)	9 [9–9] (24)	12 [12–12] (28)	<0.0001
No medication	21,613 (12.1)	157,259 (87.9)	7 [6–7] (19)	8 [8–8] (24)	<0.0001
Single medication	8712 (10.4)	75,291 (89.6)	11 [11–12] (24)	14 [14–15] (28)	<0.0001
Comedication	5904 (10.9)	48,397 (88.1)	17 [16–18] (31)	20 [19–20] (35)	<0.0001

Medication scheme	N observations (%)		Work VAS—median [95%CI] (IQR)		
	AIT	No AIT	AIT	No AIT	
All observations	17,730 (11.8)	132,002 (88.2)	6 [6–6] (18)	8 [8–8] (23)	<0.0001
No medication	10,465 (12.5)	73,024 (87.5)	4 [4–4] (15)	5 [5–6] (18)	<0.0001
Single medication	4472 (11.0)	35,997 (89.0)	8 [7–8] (18)	10 [10–10] (23)	<0.0001
Comedication	2793 (10.8)	22,981 (88.2)	12 [11–13] (27)	15 [14–15] (30)	<0.0001

Abbreviations: CI, confidence interval; IQR, interquartile range; Symptoms VAS, MASK‐air^®^ visual analogue scale assessing the severity of overall allergic symptoms on that day; Work VAS, MASK‐air^®^ visual analogue scale assessing the impact of allergic symptoms on work on that day.

In a second study (submitted), it was found that AIT was more effective on days with OAH than on those with INCS (monotherapy or comedication). AIT had little effect on Aze‐FLU.

**Limitations of the studies:** There was no differentiation between SCIT and SLIT, and between different vaccines. This was not possible due to the number of patients and the lack of information on treatments.

### 8.2 SLIT is more effective than SCIT

**FIGURE 13:**
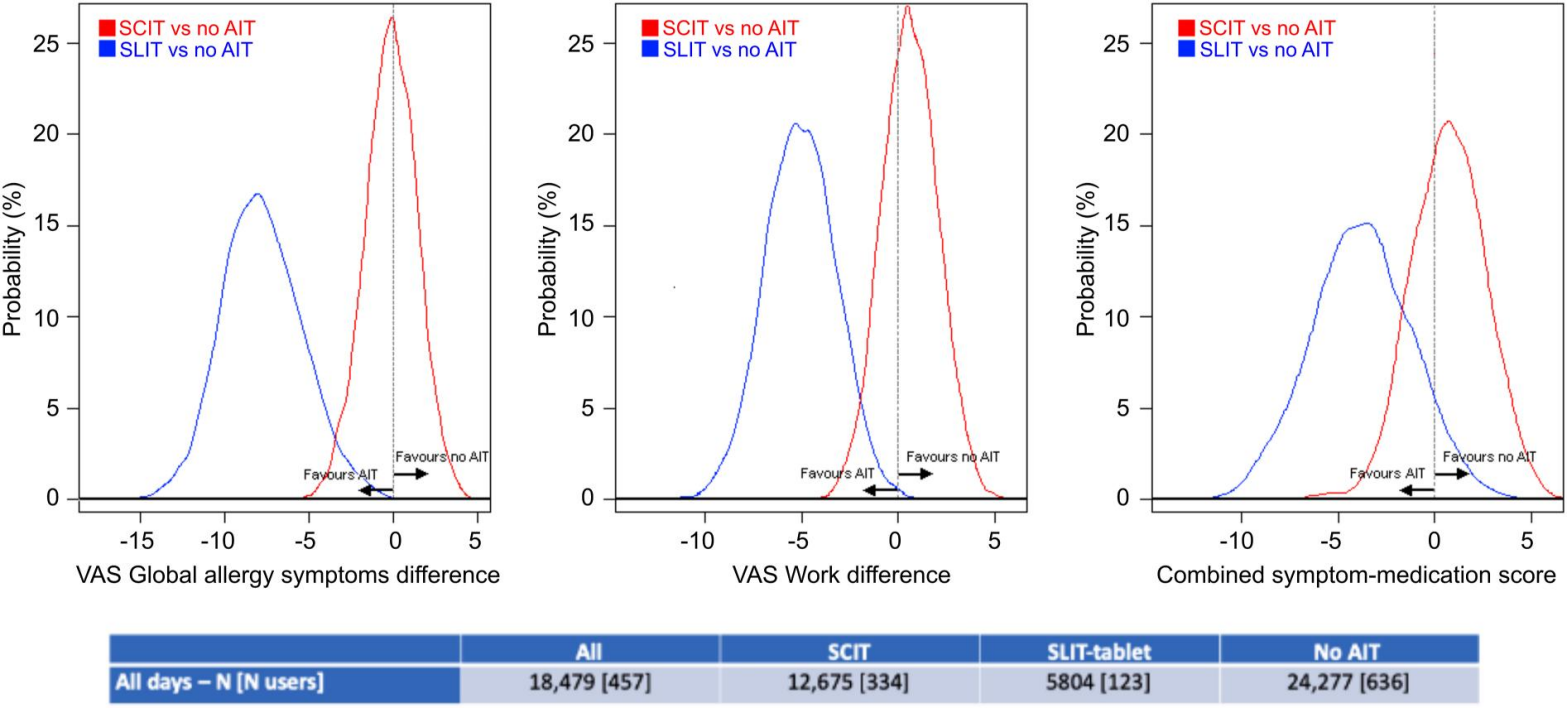
Probability distribution between SLIT, SCIT and no AIT.

The reported control of AR symptoms (VAS global allergy symptoms), work (VAS work) and CSMS was studied in users receiving sublingual AIT (SLIT) or subcutaneous AIT (SCIT), and in those with no AIT. The MASK‐air^®^ data of European users with self‐reported AR and grass pollen allergy were studied. Bayesian mixed‐effects models—with clustering by patient, country and pollen season—were analysed (Figure 13).^94^

**Limitations of the studies:** There was no differentiation between different SLIT or SCIT vaccines. This was not possible due to the number of patients.

### 8.3 AIT impact on educational activities in young people differs from pharmacotherapy

Several studies have suggested an impact of AR on academic productivity. However, large studies with direct patient data are not available. We assessed variables measuring the impact of allergies on academic performance (VAS school, WPAI + CIQ:AS impact of allergy symptoms on academic performance and percentage of hours of school lost due to allergies). Additionally, factors associated with the impact of allergic symptoms on academic productivity were assessed using multivariable mixed models.

AIT showed a strong negative association with VAS school (Table 7). On the other hand, a worse rhinitis control (CSMS) was associated with worse VAS school, higher impact on academic productivity and the percentage of hours of school missed due to allergy.^69^

**TABLE 7:** Main model of the association between VAS school and other independent variables using multi‐level mixed effects linear regression

	Association with VAS school		
	Regression coefficient	95% CI	*p*‐value
Baseline impact	1.40	0.91; 1.89	<0.001
Baseline symptoms	−0.63	−0.95; −0.30	<0.001
Male gender	−0.20	−1.47; 1.07	0.759
Age	0.00	−0.13; 0.14	0.707
AIT	−2.70	−4.41; −0.98	0.002
Oral antihistamines	0.06	−0.67; 0.79	0.119
Topical antihistamines	1.71	−0.52; 3.94	0.132
Nasal steroids	−0.85	−1.79; 0.10	0.081
Azelastine + Fluticasone	−1.36	−4.15; 1.44	0.342
Systemic steroids	5.19	1.06; 9.33	0.014^a^
Asthma medications	−0.14	−1.53; 1.25	0.842
Other rhinitis medications	−0.25	−1.53; 1.03	0.703
VAS eyes	0.19	0.17; 0.2	<0.001
VAS nose	0.37	0.36; 0.38	<0.001
VAS asthma	0.18	0.16; 0.2	<0.001

Abbreviations: CI, confidence interval; VAS, Visual Analogue Scale.

^a^ Not statistically significant after Bonferroni correction.

**Limitations of the studies:** There was no differentiation between SCIT and SLIT and between different vaccines. This was due to the number of patients and the lack of information on treatments up until 2021.

### 8.4 Adherence to SLIT appears to be better than adherence to pharmacotherapy

In a small sample (170 users), over 50% of users were fully adherent to SLIT during the pollen season.

### 8.5 Rapid relief of symptoms by SLIT

**FIGURE 14:**
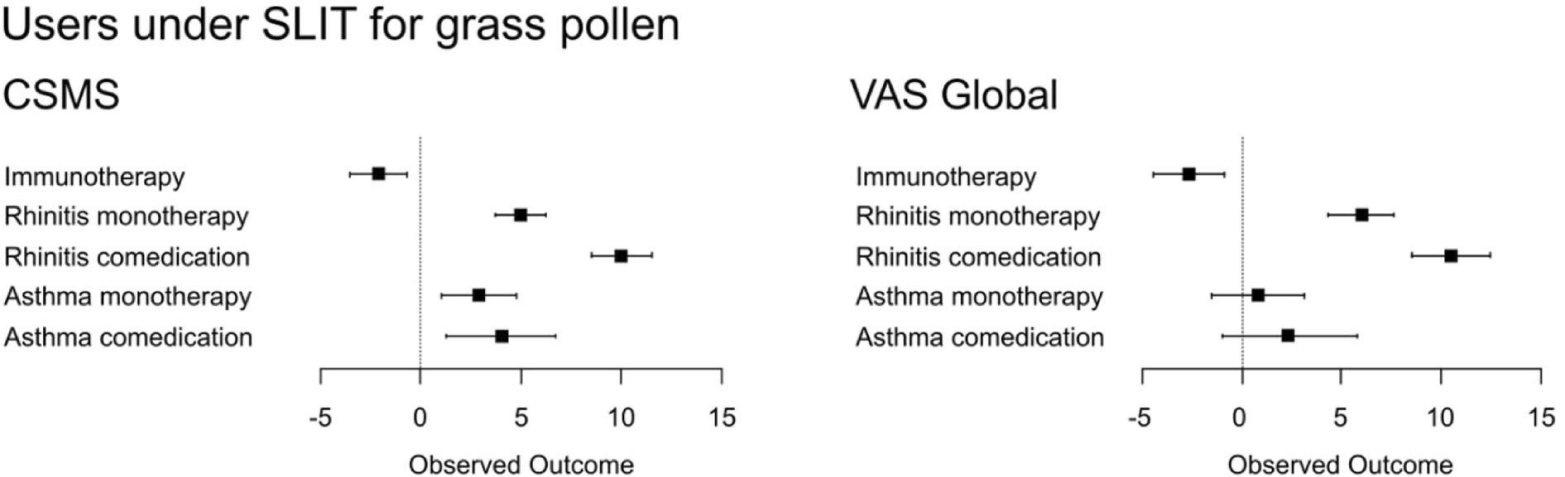
Regression coefficients.

AIT was suggested to be rapidly effective in AR.^95^ In a European cross‐sectional study, MASK‐air^®^ data were assessed in patients reporting grass pollen AIT, comparing days with AIT versus days without AIT. 2296 days from 80 patients using SCIT and 3098 days from 113 patients using SLIT were analysed. In users under SLIT, days with AIT were associated with better AR control than days without AIT, with lower CSMS and VAS global (Figure 14). Similar results were observed in sensitivity analyses. Use of AIT could not be associated with improved AR control in patients under SCIT.

AIT is proposed to be an effective treatment for AR only after weeks or months. However, the current data indicate that it is rapidly effective.^95^ As an example, rush SCIT to pollen and mites reduces skin test reactivity to allergens within days in a dose‐dependent and time‐independent manner.^96^, ^97^ These rapid clinical features cannot be explained by an adaptive immune response (immunotherapy), but may be related to rapid and short‐lasting cell downregulation (desensitisation), particularly in basophils and mast cells.^95^, ^98^ These considerations may lead to the hypothesis that SLIT may induce a rapid relief of allergic symptoms during the pollen season. However, this requires confirmation.^95^

**Limitations of the study**: Low number of users but highly‐consistent results.

## 9 EMBEDDING AEROBIOLOGY DATA IN MASK‐AIR^®^

Three EU grants were obtained to include aerobiology data in MASK‐air^®^.

### 9.1 POLLAR: Impact of air POllution on Asthma and Rhinitis (EIT health)

#### 9.1.1 The project

Allergic rhinitis (AR) is impacted by allergens and air pollution but interactions between air pollution, sleep and allergic diseases were insufficiently understood. POLLAR (Impact of air POLLution on Asthma and Rhinitis), a project of the European Institute of Innovation and Technology (EIT Health) and a demonstration project of GARD (Global Alliance against Chronic Respiratory Diseases, WHO),^99^, ^100^ used MASK‐air^®^ to investigate these relationships in Northern and Central European users in 2017 and 2018.^45^

A total of 3323 geolocated individuals (36,440 VAS‐days) were studied. Associations between uncontrolled rhinitis and pollutants were stronger during the grass pollen season.^73^ Days with uncontrolled AR increased by 25% for an interquartile range increase in ozone levels during the grass pollen season (odds ratio of 1.25 [95% CI, 1.11–1.41] in 2017 and of 1.14 [95% CI, 1.04–1.25] in 2018). A similar trend was found for particulate matter with a diameter of <2.5 µm, especially in 2017. These results suggest that the relationship between uncontrolled AR and air pollution is modified by the presence of grass pollens. They favour the inclusion of pollen and pollution data in MASK‐air^®^.

#### 9.1.2 Impact on MASK‐air^®^

A new index developed by the Finnish Meteorological Institute (FMI) is available in MASK‐air^®^ and was developed in the frame of POLLAR.^34^ On‐going transformation of the pollen allergy information support is based on new technological solutions for pollen and on the monitoring and prediction of air quality.

**FIGURE 15:**
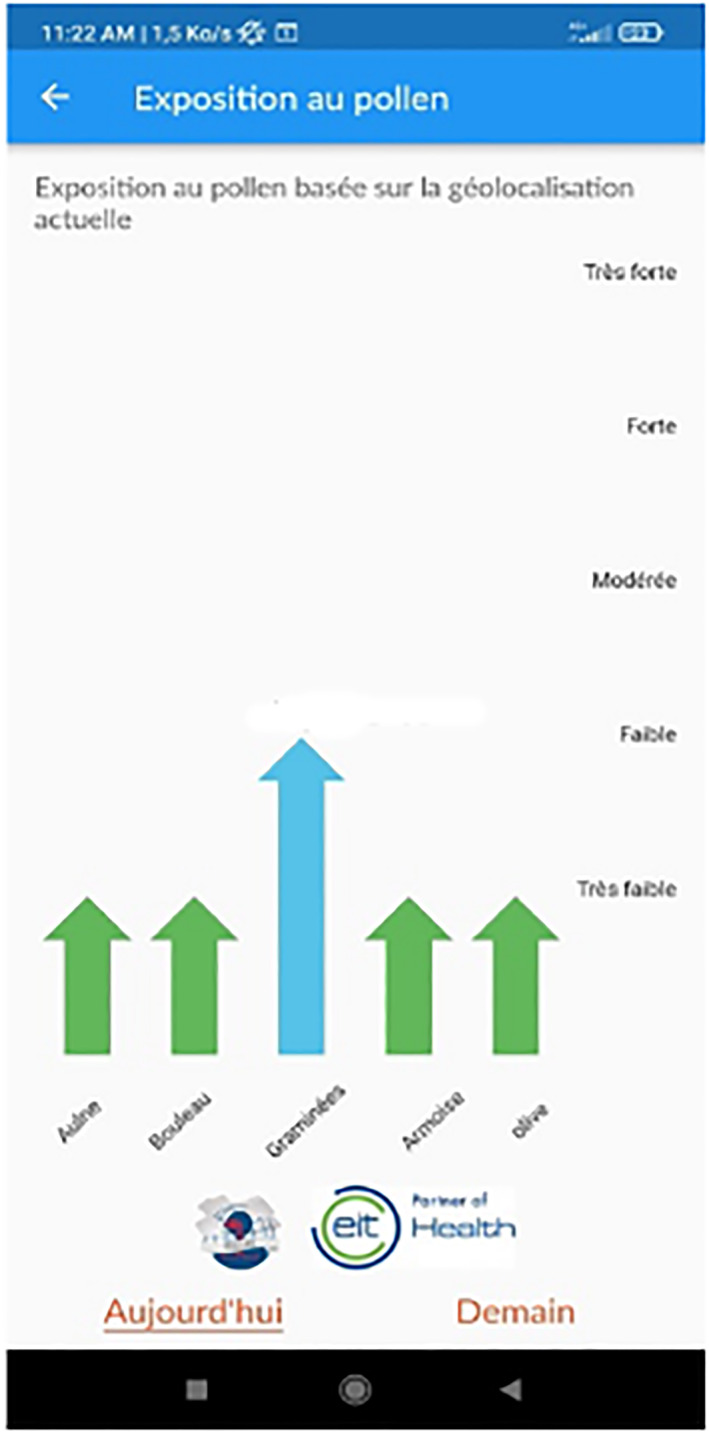
MASK‐air^®^ screen showing daily pollen counts obtained from the FMI.

Every day, in the app, the prediction of pollens (pollution available 01‐2023) is indicated for the current and for the next day (Figure 15).

### 9.2 Catalyse (Horizon Europe)

A new Horizon Europe grant began in September 2022 in order to better understand climate change and to determine how to counteract it. Pollens were selected as one of the indicators of climate change. In collaboration with the FMI and Porto University, MASK‐air^®^ will be used to correlate pollen counts with the clinical impact. For this project, preliminary methodologic studies are undergoing. They will help to better assess the impact of the pollen season on symptoms.

The integration of information technology tools for climate, weather, air pollution and aerobiology in mobile Health app will enable the development of an alert system. Citizens will thus be informed about personal environmental threats, which may also be linked to indicators of Planetary Health and sustainability.

## 10 ACCEPTABILITY OF MASK‐AIR^®^ BY PATIENTS

Many patients do not understand the needs and benefits of mHealth and may worry about data privacy. Thus, the uptake of mHealth is slow. On the other hand, too many patients over‐rely on internet‐based information and on untested mHealth solutions. This attitude may have dangerous implications since patients may receive an incorrect diagnosis or management strategy.

A qualitative study was carried out by MADOPA in 2016 for MASK to better understand the patients' needs and expectations (Table 8).

**TABLE 8:** Patients' expectations

Patients' expectations	Existing feature	To be added	
		Feature	Expected
Advice to modify the treatment	Simple advice exists in line with the GDPR		Available
		More sophisticated advice will be ready with class 2 MDR	06–2023
Pollen and pollution		POLLAR	03–2021
Visualisation of control and medications	Existing but poorly found by patients and physicians	More user friendly and better information	Available
Help science to better understand the disease in order to get future benefits	Existing		

## 11 IMPLICATIONS OF DIRECT PATIENT MASK‐AIR^®^ DATA AND RESEARCH NEEDS

### 11.1 Treatment of rhinitis alone versus rhinitis and asthma

Rhinitis alone and rhinitis and asthma represent two different diseases with differences in genetic background,^90^, ^101^ allergen sensitisation,^84^, ^88^, ^102^ inflammation (blood eosinophils),^84^ age of onset,^84^, ^102^ prevalence of conjunctivitis,^84^, ^85^ severity of rhinitis^85^ and response to treatment. It is important for guidelines to reflect these findings. However, available RCTs will not find differences without re‐analysing the data on file. Moreover, since most patients in RCTs have severe symptoms, differentiating between the two phenotypes may not be easy.

### 11.2 Value‐Added Medicines: PRN rather than long‐term (not for CRS)

Drug repurposing is one of the major fields of Value‐Added Medicines.^103^, ^104^, ^105^, ^106^ Often based on direct patient data, it involves the investigation of existing drugs for new therapeutic purposes that address healthcare needs. Several unmet needs in AR could be improved by drug repurposing. This could be game‐changing for disease management. The current AR medications usually follow a continuous long‐term treatment, and medication registration is based on RCTs carried out for 14 days (at least) with adherence ≥70%.

A new way of treating AR is to propose an alternative to the classical continuous treatment, that of an as‐needed treatment depending on symptoms.^107^ This debate has been resolved in asthma by four trials (two RCTs of the SYGMA project and two real‐life studies: Novel START and the PRACTICAL trial) (for review see^108^). These trials have shown the benefits of substituting short‐acting β‐agonists with budesonide‐formoterol as a rescue medication in mild asthma patients.

### 11.3 The step‐up‐step‐down strategy needs to be reconsidered in some patients using mHealth tools

Guidelines for rhinitis or asthma propose a step‐up and step‐down strategy. However, MASK‐air^®^ studies show that, in most cases, when patients are uncontrolled, they use any medication and not the appropriate one. This suggests that a very simple approach needs to be considered for mild symptoms and for when they are getting worse.

Moreover, in AR, around 10% of patients are adherent and an alternative approach is needed for these cases with a defined step‐up‐step‐down strategy.^107^

Overall, there is an urgent need to develop an electronic clinical decision support system for patients who are not controlled with first‐line treatments and who need better assessment.^30^

### 11.4 mHealth biomarkers in rhinitis for patient stratification and follow‐up

Biomarkers that reflect biological processes are essential for monitoring the health of patients. They include clinical signs, biological assays, mHealth outcomes, genomic indices and others that can be objectively measured and used as indicators of pathophysiological processes.^109^

The new ARIA‐EAACI CSMS is a **validated, real‐life, digitally‐enabled, patient‐centred biomarker for any treatment, particularly AIT.** It was found to be applicable to different languages and cultures (Table 9).

**TABLE 9:** Implications of the allergy‐CSMS

Clinical practice Indication of a treatment in stratified patients Follow up of a treatment and early stopping rule Follow up of a treatment and regular review of efficacy Follow up of patients after the treatment is stopped If the patient relapses, follow up of patients after reintroduction of the treatment to assess the benefits of a next course RCTs, currently as an exploratory end point but requiring validation as a primary end point Observational studies that will triangulate RCTs and make a link with clinical practice Patients' centred real‐life data Challenges (allergen) to better relate the results with real life

**FIGURE 16:**
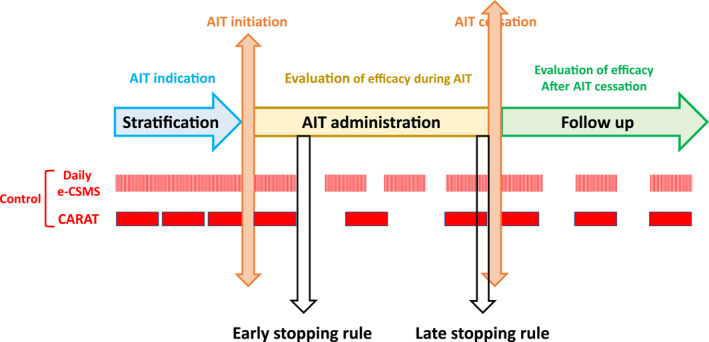
Applicability of mHealth biomarkers in AIT.

**By analogy with diabetes,** ^110^ **two types of mHealth biomarkers can be defined** in rhinitis (Figure 16):

- Daily monitoring of the control (analogous to glycemia measurement): ARIA‐EAACI CSMS
- Longer‐term monitoring using control scores (analogous to Hb1AC measurement): CARAT.^32^, ^43^, ^111^ For this approach to extend to asthma, a CSMS assessing short‐term asthma control needs to be developed.

In AIT, the allergy‐CSMS can be used to (i) stratify patients (uncontrolled days during the allergen exposure, e.g., pollen season, despite guideline‐based treatment in patients adherent to treatment), (ii) propose an early stopping rule, (iii) follow the patient during the treatment and (iv) follow the patient during the after‐cessation follow‐up (Figure 16). However, a dual approach can be proposed combining the daily allergy‐CSMS with a control test for allergic diseases assessing at least 1 month of survey.

### 11.5 Optimisation of shared decision making

In shared decision making (SDM), both the patient and the physician contribute to the medical decision‐making process, placing the patient at the centre of the decision paradigm.^112^, ^113^ An innovation in SDM is the use of mHealth evidence‐based tools that can inform patient decisions based on a guided self‐management plan proposed by their healthcare professional.^114^

In MASK‐air^®^, an e‐CDSS has been devised.^30^ However, it has not yet been implemented because it needed an MDR Class IIa accreditation (January 2022) after ethical approval. mHealth biomarkers for daily and long‐term control will help SDM.

In daily practice, MASK‐air^®^ can be used for the optimisation of SDM since the physician can obtain the daily information of medication and control when the patient is consulting.^82^

### 11.6 School, work and economic impacts

In MASK‐air^®^, several outcomes have an economic impact: EQ‐5D VAS, Work VAS and WPAI‐AS.^48^, ^53^, ^59^ Combining these data, a plan is being devised to ascribe an economic impact to CSMS.

### 11.7 Next‐generation digitally‐enabled, patient‐centred care pathways

Large observational implementation studies are needed to triangulate RCTs. The results of MASK‐air^®^ have allowed the development of next‐generation guidelines assessing the recommendations of GRADE guidelines in rhinitis and asthma using real‐world evidence.^115^ However, these recommendations were based on a consensus.

The NextGen ARIA guidelines 2023 will be developed with real patient data, analysis of new data and new methods facilitating the process of prioritising questions and health outcomes in guideline development. This will support the creation of trustworthy guidelines following a structured plan (in collaboration with HJ Schünemann and J Brozek):^116^, ^117^, ^118^, ^119^, ^120^

- Question prioritisation including, if possible, (i) differentiation between rhinitis and asthma + rhinitis, (ii) comedication and (iii) AIT.
- Evidence‐based analysis including meta‐analyses.
- Integration of direct patient data including MASK‐air^®^.
- GRADE Evidence to Decision (EtD) frameworks.^121^, ^122^
- Panel education, for example, INGUIDE (International Guideline Development Credentialling & Certification Programme, a comprehensive, evidence‐based, and up‐to‐date training programme for guideline recommendation and development) (https://inguide.org).

It is expected that the NextGen ARIA guidelines 2023 will be developed in collaboration with OECD, Fraunhofer and several scientific and patient organisations.

### 11.8 Climate change and planetary health

The digital transformation of health and care to sustain Planetary Health was initiated by (i) the MASK proof‐of‐concept for airway diseases‐POLLAR symposium under the auspices of Finland's Presidency of the EU, 2019, (ii) MACVIA‐France, (iii) the Global Alliance against Chronic Respiratory Diseases (GARD, WH0) demonstration project and (iv) the Reference Site Collaborative Network of the European Innovation Partnership on Active and Healthy Ageing.^123^

In December 2019, a conference entitled ‘Europe That Protects: Safeguarding Our Planet, Safeguarding Our Health’ was held in Helsinki. It was co‐organised by the Finnish Institute for Health and Welfare, the Finnish Environment Institute and the European Commission, under the auspices of Finland's Presidency of the EU. As a side event, a symposium organised as the final POLLAR (Impact of air POLLution on Asthma and Rhinitis) meeting explored the digital transformation of health and care to sustain planetary health in airway diseases. The Finnish Allergy Programme collaborates with MASK (Mobile Airways Sentinel NetworK) and can be considered as a proof‐of‐concept to impact Planetary Health.

### 11.9 Change management and political agenda for digitally‐enabled, patient‐centred care pathways

ARIA has evolved from a guideline using the best approach^124^ to integrated care pathways using mobile technology in patients with allergic rhinitis (AR) and asthma multimorbidity.^125^ The proposed next phase of ARIA is change management.^126^ The aim of this phase is to provide an active and healthy life to patients with rhinitis and to those with asthma multimorbidity across the lifecycle, irrespective of their sex or socioeconomic status. The aim is to reduce health and social inequities incurred by the disease. ARIA will follow the 8‐step model of Kotter (i) to assess and implement the effect of rhinitis on asthma multimorbidity and (ii) to propose NextGen guidelines. These guidelines will need to be presented to regulators and payers and this may be possible through the OECD.

## AUTHOR CONTRIBUTIONS

Jean Bousquet is the chair of MASK‐air^®^. Josep M. Anto, Anna Bedbrook, Sinthia Bosnic‐Anticevich, Wienczyslawa Czarlewski, Joao A Fonseca, Tari Haahtela, Ludger Klimek, Oliver Pfaar, Piotr Kuna, Maciej Kupczyk, Frederico S. Regateiro, Boleslaw Samolinski, Bernardo Sousa‐Pinto, Arunas Valiulis, Arzu Yorgancioglu and Torsten Zuberbier are in the MASK‐air^®^ steering committee. Govert De Vries, Daniel Laune, Yann Micheli, Eve Mathieu‐Dupas, Michiel Van Eerd and Frédéric Viart are involved in IT and MASK‐air management. All remaining authors participated in data collection and writing – review & editing. All authors have reviewed and approved the manuscript.

## CONFLICTS OF INTEREST

**Dr. Bosnic‐Anticevich** reports grants from TEVA, personal fees from TEVA, personal fees from TEVA, personal fees from AstraZeneca, personal fees from AstraZeneca, personal fees from Boehringer Ingelheim, personal fees from Boehringer Ingelheim, personal fees from GSK, personal fees from Sanofi, personal fees from Mylan, outside the submitted work. **Dr. Bousquet** reports personal fees from Cipla, Menarini, Mylan, Novartis, Purina, Sanofi‐Aventis, Teva, Uriach, other from KYomed‐Innov, other from Mask‐air‐SAS, outside the submitted work. **Dr. Cardona** reports personal fees from ALK, personal fees from Allergopharma, personal fees from GSK, grants from Thermofisher, outside the submitted work. **Dr. Cecchi** reports personal fees from Thermofisher, personal fees from Sanofi, personal fees from Astra Zeneca, personal fees from Novartis, outside the submitted work. **Dr. Chu** reports AAAAI Foundation Faculty Development Awardee. **Dr. Cruz** reports personal fees from AstraZeneca, personal fees from Chiesi, personal fees from GSK, personal fees from SANOFI, personal fees from Novartis, personal fees from Boehringer Ingelheim, personal fees from Glennmark, personal fees from Eurofarma, personal fees from Abdi Ibrahim, personal fees from CROSSJECT, outside the submitted work. **Dr. Del Giacco** reports grants from AstraZeneca, grants from GSK, grants from Novartis, grants from Sanofi, outside the submitted work. **Dr. Devillier** reports personal fees from ALK Abello, personal fees and non‐financial support from Boehringer Ingelheim, personal fees from Chiesi, personal fees and non‐financial support from Astra Zeneca, personal fees from GlaxoSmithKline, personal fees from Menarini, personal fees from Novartis, personal fees and non‐financial support from Stallergenes, personal fees from Sanofi, outside the submitted work. **Dr. Fonseca** reports being co‐founder of an SME that develops mHealth technologies, such as digital biomarkers and has the copyright of the CARAT anda CARATkids PROM. **Dr. Haahtela** reports personal fees from Orion Pharma, outside the submitted work. **Dr. Ivancevich** reports personal fees from Laboratorios Casasco, personal fees from Bago Bolivia, personal fees from Abbott Ecuador, personal fees from Faes Farma, outside the submitted work. **Dr. Jutel** reports personal fees from ALK‐Abello, personal fees from Allergopharma, personal fees from Stallergenes, personal fees from Anergis, personal fees from Allergy Therapeutics, personal fees from Leti, personal fees from HAL, during the conduct of the study; personal fees from GSK, personal fees from Novartis, personal fees from Teva, personal fees from Takeda, personal fees from Chiesi, outside the submitted work. **Dr. Klimek** reports grants and personal fees from Allergopharma, grants and personal fees from Viatris, personal fees from HAL Allergie, personal fees from ALK Abelló, grants and personal fees from LETI Pharma, grants and personal fees from Stallergenes, grants from Quintiles, grants and personal fees from Sanofi, grants from ASIT biotech, grants from Lofarma, personal fees from Allergy Therapeut., grants from AstraZeneca, grants and personal fees from GSK, grants from Inmunotek, personal fees from Cassella med, personal fees from Novartis, personal fees from Regeneron Pharmaceuticals, personal fees from ROXALL Medizin GmbH, outside the submitted work; and Membership: AeDA, DGHNO, Deutsche Akademie für Allergologie und klinische Immunologie, HNO‐BV, GPA, EAACI. **Dr. Kraxner** reports personal fees from Mylan/Viatris, personal fees from Sanofi, outside the submitted work. **Dr. Kritikos** reports personal fees from AstraZeneca, outside the submitted work. **Dr. Kuna** reports personal fees from Adamed, personal fees from Berlin Chemie Menarini, personal fees from AstraZeneca, personal fees from Boehringer Ingelheim, personal fees from Celon Pharma, personal fees from Polpharma, personal fees from Teva, personal fees from Novartis, personal fees from Glenmark, personal fees from Zentiva, outside the submitted work. **Dr. Kupczyk** reports personal fees from Astra Zeneca, personal fees from GSK, personal fees from Adamed, personal fees from Polpharma, personal fees from Celon Pharma, personal fees from Berlin Chemie Menarini, personal fees from Novartis, personal fees from Nexter Allergopharma, personal fees from Teva, personal fees from Chiesi, personal fees from Zentiva, personal fees from Sanofi Aventis, personal fees from Pfizer, personal fees from Abbvie, personal fees from Lekam, outside the submitted work. **Dr. Kvedariene** reports other from NORAMEDA, outside the submitted work. **Dr. Larenas Linnemann** reports personal fees from ALK, Allakos, Amstrong, Astrazeneca national and global, Chiesi, DBV Technologies, Grunenthal, Grin, GSK national and global, Viatris, Menarini, MSD, Novartis, Pfizer, Sanofi, Siegfried, UCB, Carnot, grants from Abbvie, Bayer, Lilly, Sanofi, Astrazeneca, Lilly, Pfizer, Novartis, Circassia, UCB, GSK, Purina institute., outside the submitted work. **Dr. Louis** reports grants from GSK, Chiesi and AZ and adboard and lecture fees from AZ, GSK, Chiesi. **Dr. Mösges** reports personal fees from ALK, grants from ASIT biotech, personal fees from allergopharma, personal fees from Allergy Therapeutics, grants and personal fees from Bencard, grants from Leti, grants, personal fees and non‐financial support from Lofarma, non‐financial support from Roxall, grants and personal fees from Stallergenes, grants from Optima, personal fees from Friulchem, personal fees from Hexal, personal fees from Servier, personal fees from Klosterfrau, non‐financial support from Atmos, personal fees from Bayer, non‐financial support from Bionorica, personal fees from FAES, personal fees from GSK, personal fees from MSD, personal fees from Johnson&Johnson, personal fees from Meda, personal fees and non‐financial support from Novartis, non‐financial support from Otonomy, personal fees from Stada, personal fees from UCB, non‐financial support from Ferrero, grants from BitopAG, grants from Hulka, personal fees from Nuvo, grants and personal fees from Ursapharm, personal fees from Menarini, personal fees from Mundipharma, personal fees from Pohl‐Boskamp, grants from Inmunotek, grants from Cassella‐med GmbH & Co. KG, personal fees from Laboratoire de la Mer, personal fees from Sidroga, grants and personal fees from HAL BV, personal fees from Lek, personal fees from PRO‐AdWise, personal fees from Angelini Pharma, grants and non‐financial support from JGL, outside the submitted work. **Dr. Mullol** reports personal fees and other from SANOFI‐GENZYME & REGENERON, personal fees and other from NOVARTIS, grants and personal fees from VIATRIS, grants and personal fees from URIACH Group, personal fees from Mitsubishi‐Tanabe, personal fees from Menarini, personal fees from UCB, personal fees from AstraZeneca, grants and personal fees from GSK, personal fees from MSD, outside the submitted work. **Dr. Okamoto** reports personal fees from Torii pharmaceutical Co., LTD., personal fees from Tanabe‐Mitsubishi Pharmaceutical Co., Ltd., personal fees from Kirin Holdings Co., Ltd., personal fees from Novartis Co., Ltd., personal fees from Allergologisk Laboratorium København, personal fees from Shionogi Co., Ltd., outside the submitted work. **Dr. Papadopoulos** reports personal fees from NOVARTIS, personal fees from NUTRICIA, personal fees from HAL, personal fees from MENARINI/FAES FARMA, personal fees from SANOFI/REGENERON, personal fees from MYLAN/MEDA, personal fees from BIOMAY, personal fees from AstraZeneca, personal fees from GSK, personal fees from MSD, personal fees from ASIT BIOTECH, personal fees from Boehringer Ingelheim, grants from CAPRICARE, grants from Gerolymatos Int, grants from NUTRICIA, personal fees from MEDCARE, personal fees from ALK, personal fees from OM PHARMA, from ABBOTT, outside the submitted work. **Dr. Pfaar reports** grants and personal fees from ALK‐Abelló, grants and personal fees from Allergopharma, grants and personal fees from Stallergenes Greer, grants and personal fees from HAL Allergy Holding B.V./HAL Allergie GmbH, grants and personal fees from Bencard Allergie GmbH/Allergy Therapeutics, grants and personal fees from Lofarma, grants from Biomay, grants from Circassia, grants and personal fees from ASIT Biotech Tools S.A., grants and personal fees from Laboratorios LETI/LETI Pharma, personal fees from MEDA Pharma/MYLAN, grants and personal fees from Anergis S.A., personal fees from Mobile Chamber Experts (a GA2LEN Partner), personal fees from Indoor Biotechnologies, grants and personal fees from GlaxoSmithKline, personal fees from Astellas Pharma Global, personal fees from EUFOREA, personal fees from ROXALL Medizin, personal fees from Novartis, personal fees from Sanofi‐Aventis and Sanofi‐Genzyme, personal fees from Med Update Europe GmbH, personal fees from streamedup! GmbH, grants from Pohl‐Boskamp, grants from Inmunotek S.L., personal fees from John Wiley and Sons, AS, personal fees from Paul‐Martini‐Stiftung (PMS), personal fees from Regeneron Pharmaceuticals Inc., personal fees from RG Aerztefortbildung, personal fees from Institut für Disease Management, personal fees from Springer GmbH, grants and personal fees from AstraZeneca, personal fees from IQVIA Commercial, personal fees from Ingress Health, personal fees from Wort&Bild Verlag, personal fees from Verlag ME, personal fees from Procter&Gamble, outside the submitted work. **Dr. Quirce** reports personal fees and non‐financial support from GSK, personal fees and non‐financial support from AstraZeneca, personal fees and non‐financial support from Sanofi, personal fees and non‐financial support from Novartis, personal fees and non‐financial support from Mundipharma, personal fees and non‐financial support from Teva, personal fees and non‐financial support from Allergy Therapeutics, outside the submitted work. **Dr. Roche** reports grants and personal fees from Boehringer Ingelheim, grants and personal fees from Novartis, grants and personal fees from GSK, personal fees from AstraZeneca, personal fees from Chiesi, grants and personal fees from Pfizer, personal fees from Sanofi, personal fees from Zambon, personal fees from MSD, personal fees from Austral, outside the submitted work. **Dr. Samoliński** reports personal fees from Polpharma, personal fees from Viatris, grants and personal fees from AstraZeneca, personal fees from TEVA, personal fees from patient ombudsman, personal fees from Polish Allergology Society, grants from GSK, outside the submitted work. **Dr. Sastre** reports grants and personal fees from SANOFI, personal fees from GSK, personal fees from NOVARTIS, personal fees from ASTRA ZENECA, personal fees from MUNDIPHARMA, personal fees from FAES FARMA, outside the submitted work. **Dr. Serpa** reports personal fees and other from Novartis, personal fees from Takeda, personal fees from CSL Behring, personal fees from GSK, other from Astra Zeneca, outside the submitted work. **Dr. Taborda‐Barata** reports personal fees from AstraZeneca, personal fees from Diater, outside the submitted work. **Dr. Toppila‐Salmi** reports grants from GSK, personal fees from AstraZeneca, personal fees from ALK Abello, personal fees from Roche, personal fees from Novartis, personal fees from Sanofi Pharma, outside the submitted work. **Dr. Tsiligianni** reports grants from GSK Hellas, Astra Zeneca Hellas,Boehringer Ingelheim, personal fees from Novartis, Johnson and Johnson, Boehringer Ingelheim, Chiesi, Astra Zeneca, outside the submitted work. **Dr. Zidarn** reports personal fees from TAKEDA, outside the submitted work. **Dr. Zuberbier** reports grants and personal fees from Novartis, grants and personal fees from Henkel, personal fees from Bayer, personal fees from FAES, personal fees from Astra Zeneca, personal fees from AbbVie, personal fees from ALK, personal fees from Almirall, personal fees from Astellas, personal fees from Bayer, personal fees from Bencard, personal fees from Berlin Chemie, personal fees from FAES, personal fees from Hal, personal fees from Leti, personal fees from Mesa, personal fees from Menarini, personal fees from Merck, personal fees from MSD, personal fees from Novartis, personal fees from Pfizer, personal fees from Sanofi, personal fees from Stallergenes, personal fees from Takeda, personal fees from Teva, personal fees from UCB, personal fees from Henkel, personal fees from Kryolan, personal fees from L'Oreal, outside the submitted work; and Organizational affiliations: Commitee member: WHO‐Initiative ‘Allergic Rhinitis and Its Impact on Asthma’ (ARIA), Member of the Board: German Society for Allergy and Clinical Immunology (DGAKI), Head: European Centre for Allergy Research Foundation (ECARF), President: Global Allergy and Asthma European Network (GA2LEN), Member: Committee on Allergy Diagnosis and Molecular Allergology, World Allergy Organization (WAO). The other authors have nothing to disclose, outside the submitted work.
